# CAT: a computational anatomy toolbox for the analysis of structural MRI data

**DOI:** 10.1093/gigascience/giae049

**Published:** 2024-08-05

**Authors:** Christian Gaser, Robert Dahnke, Paul M Thompson, Florian Kurth, Eileen Luders, the Alzheimer's Disease Neuroimaging Initiative

**Affiliations:** Department of Psychiatry and Psychotherapy, Jena University Hospital, 07747 Jena, Germany; Department of Neurology, Jena University Hospital, 07747 Jena, Germany; German Center for Mental Health (DZPG), Germany; Department of Psychiatry and Psychotherapy, Jena University Hospital, 07747 Jena, Germany; Department of Neurology, Jena University Hospital, 07747 Jena, Germany; German Center for Mental Health (DZPG), Germany; Imaging Genetics Center, Stevens Neuroimaging & Informatics Institute, Keck School of Medicine, University of Southern California, Los Angeles, CA 90033, USA; School of Psychology, University of Auckland, Auckland 1142, New Zealand; Departments of Neuroradiology and Radiology, Jena University Hospital, 07747 Jena, Germany; School of Psychology, University of Auckland, Auckland 1142, New Zealand; Department of Women's and Children's Health, Uppsala University, 75237 Uppsala, Sweden; Swedish Collegium for Advanced Study (SCAS), 75236 Uppsala, Sweden; Laboratory of Neuro Imaging, School of Medicine, University of Southern California, Los Angeles, CA 90033, USA

**Keywords:** brain, computational anatomy, longitudinal, morphometry, SPM12, CAT12, MRI, ROI, VBM, cortical thickness, cortical surface, cortical folding, Alzheimer’s disease

## Abstract

A large range of sophisticated brain image analysis tools have been developed by the neuroscience community, greatly advancing the field of human brain mapping. Here we introduce the Computational Anatomy Toolbox (CAT)—a powerful suite of tools for brain morphometric analyses with an intuitive graphical user interface but also usable as a shell script. CAT is suitable for beginners, casual users, experts, and developers alike, providing a comprehensive set of analysis options, workflows, and integrated pipelines. The available analysis streams—illustrated on an example dataset—allow for voxel-based, surface-based, and region-based morphometric analyses. Notably, CAT incorporates multiple quality control options and covers the entire analysis workflow, including the preprocessing of cross-sectional and longitudinal data, statistical analysis, and the visualization of results. The overarching aim of this article is to provide a complete description and evaluation of CAT while offering a citable standard for the neuroscience community.

## Background

The study of the human brain using neuroimaging methods is still in its infancy, but rapid technical advances in image acquisition and processing are enabling ever more refined characterizations of its micro- and macro-structure. Enormous efforts, for example, have been made to map differences between groups (e.g., young vs. old, diseased vs. healthy, male vs. female), to capture changes over time (e.g., from infancy to old age, in the framework of neuroplasticity, as a result of a clinical intervention), or to assess correlations of brain attributes (e.g., measures of length, volume, shape) with behavioral, cognitive, or clinical parameters. Popular neuroimaging software packages include tools for analysis and visualization, such as SPM (RRID:SCR_007037) [1], FreeSurfer (RRID:SCR_001847) [2], the Human Connectome Workbench [3], FSL (RRID:SCR_002823) [4], BrainVISA [5], CIVET [6], or the LONI tools [7], just to name a few.

SPM (short for Statistical Parametric Mapping) is one of the most frequently used software packages, which works with MATLAB (RRID:SCR_001622) as well as Octave. Its library of accessible and editable scripts provides an ideal basis to extend the repertoire of preprocessing and analysis options. Over the years, SPM has inspired developers to create powerful tools that use SPM’s functionality and interface [8]. These tools are more than just extensions of SPM, offering a comprehensive range of cutting-edge options across the whole analysis spectrum, from the initial data processing to the final visualization of the statistical effects.

One such tool is CAT (short for Computational Anatomy Toolbox [9]). CAT constitutes a significant step forward in the field of human brain mapping by adding sophisticated methods to process and analyze structural brain magnetic resonance imaging (MRI) data using voxel-, surface-, and region-based approaches. CAT is available as a collection of accessible scripts, with an intuitive user interface, and uses the same batch editor as SPM, which allows for a seamless integration with SPM workflows and other toolboxes, such as Brainstorm [10] and ExploreASL [11]. Not only does this enable beginners and experts to run complex state-of-the-art structural image analyses within the SPM environment, but it will also provide advanced users as well as developers the much-appreciated option to incorporate a wide range of functions in their own customized workflows and pipelines.

## Findings

### Concept of CAT

CAT12 is the current version of the CAT software and runs in MATLAB (MathWorks) or as a standalone version with no need for a MATLAB license. It was originally designed to work with SPM12 [12] and is compatible with MATLAB versions 7.4 (R2007a) and later. No additional software or toolbox is required. The latest version of CAT can be downloaded here: [9]. The precompiled standalone version for Windows, Mac, or Linux operating systems can be downloaded here: [13]. All steps necessary to install and run CAT are documented in the user manual [14] and in the complementary online help, which can be accessed directly via CAT’s help functions. The CAT software is free but copyrighted and distributed under the terms of the GNU General Public License, as published by the Free Software Foundation.

**Figure 1:**
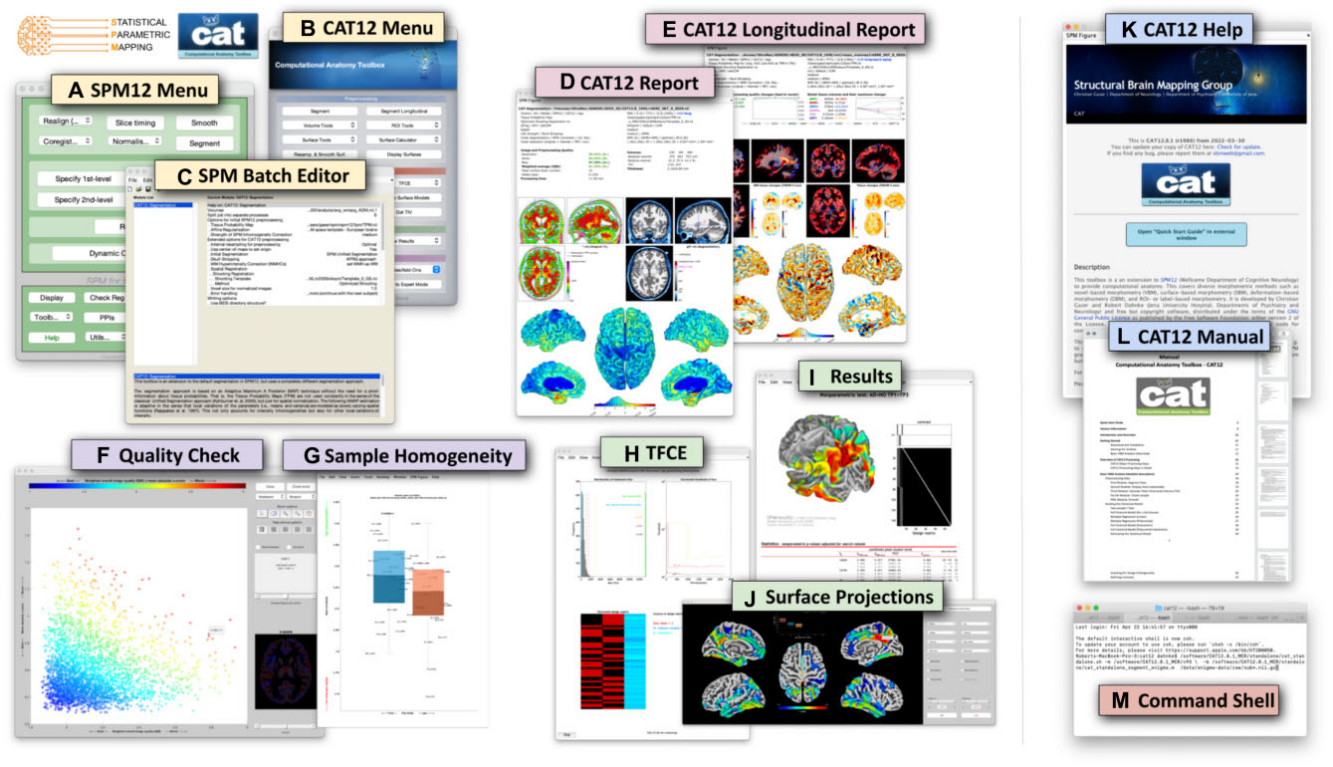
Elements of the graphical user interface. The SPM menu (A) and CAT menu (B) allow access to the (C) SPM batch editor to control and combine a variety of functions. At the end of the processing stream, cross-sectional and longitudinal outputs are summarized in a brain-specific 1-page report (D, E). In addition, CAT provides options to check image quality (F) and sample homogeneity (G) to allow outliers to be removed before applying the final statistical analysis, including threshold-free cluster enhancement—TFCE (H); the numerical and graphical output can then be retrieved (I), including surface projections (J). For beginners, there is an interactive help (K) as well as a user manual (L). For experts, command line tools (M) are available under Linux and MacOS.

CAT can be started through SPM, from the MATLAB command window, from a shell, or as a standalone version. Except when called from the command shell (CAT is fully scriptable), a user interface will appear (see Fig. 1), allowing easy access to all analysis options and most additional functions. In addition, a graphical output window will display the interactive help to get started. This interactive help will be replaced by the results of the analyses (i.e., in that same window) but can always be called again via the user interface.

### Computational morphometry

**Figure 2:**
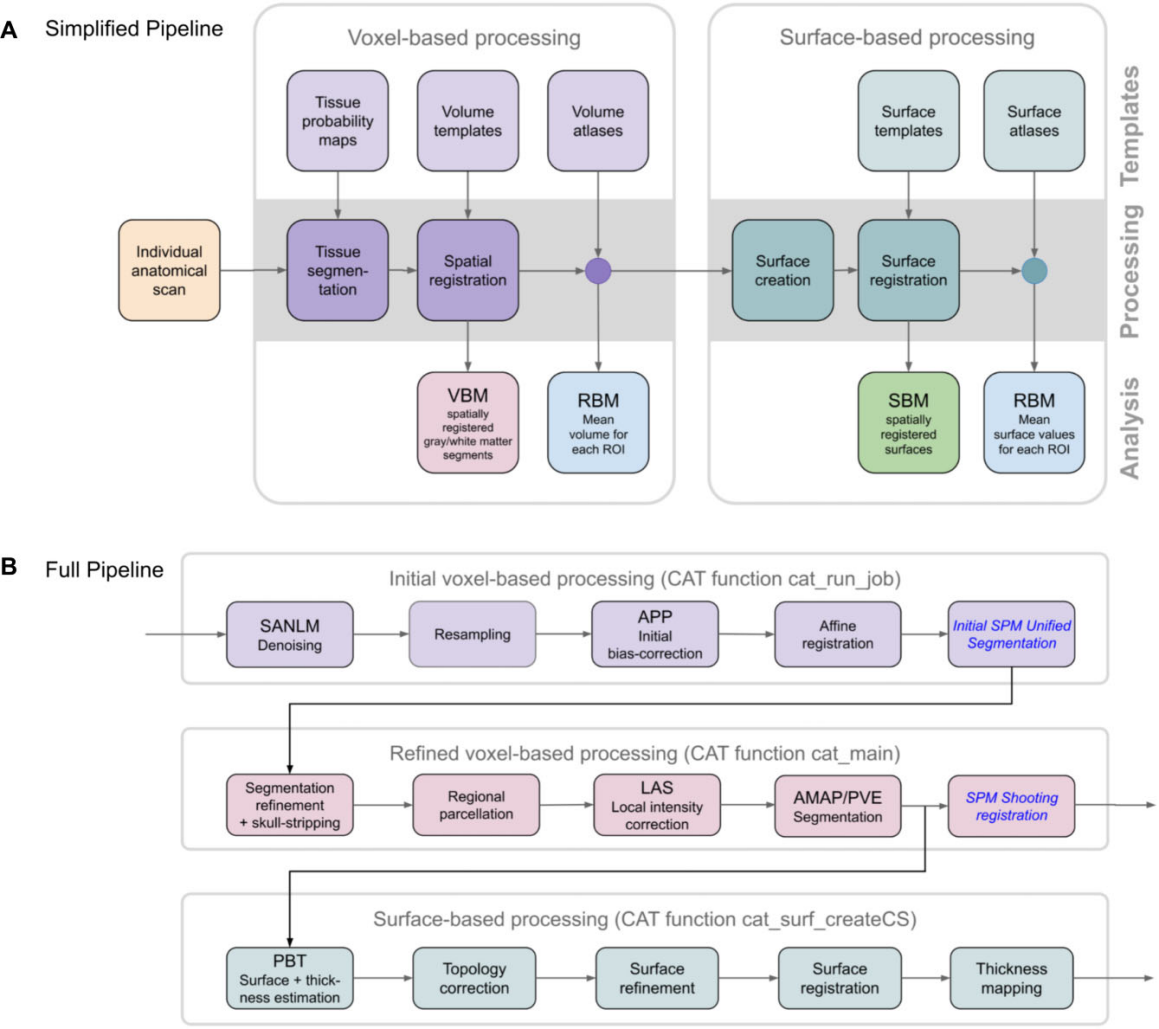
Main processing streams. (A) Simplified pipeline: image processing in CAT can be separated into a mandatory voxel-based processing stream and an optional subsequent surface-based processing stream. Each stream requires different templates and atlases and, in addition, tissue probability maps for the voxel-based stream. The voxel-based stream consists of 2 main modules—for tissue segmentation and spatial registration—resulting in spatially registered (and modulated) gray matter/white matter segments, which provides the basis for *voxel-based morphometry* (VBM). The surface-based stream also consists of 2 main modules—for surface creation and registration—resulting in spatially registered surface maps, which provide the basis for *surface-based morphometry* (SBM). Both streams also include an optional module each to analyze *regions of interest* (ROIs) resulting in ROI-specific mean volumes (mean surface values, respectively). This provides the basis for *region-based morphometry* (RBM). (B) Detailed pipeline: to illustrate the differences from SPM, the CAT pipeline is detailed with its individual processing steps. The SPM methods used are shown in blue and italic font: images are first denoised by a spatially adaptive nonlocal means (SANLM) filter [15] and resampled to an isotropic voxel size. After applying an initial bias correction to facilitate the affine registration, SPM’s unified segmentation [16] is used for the skull stripping and as a starting estimate for the adaptive maximum *a posteriori* (AMAP) segmentation [17] with partial volume estimation (PVE) [18]. In addition, SPM’s segmentation is used to locally correct image intensities. Finally, the outcomes of the AMAP segmentation are registered to the MNI template using SPM’s shooting registration. The outcomes of the AMAP segmentation are also used to estimate cortical thickness and the central surface using a projection-based thickness (PBT) method [19]. More specifically, after repairing topology defects [20], central, pial, and white matter surface meshes are generated. The individual left and right central surfaces are then registered to the corresponding hemisphere of the FreeSurfer template using a 2D version of the DARTEL approach [21]. In the final step, the pial and white matter surfaces are used to refine the initial cortical thickness estimate using the FreeSurfer thickness metric [22, 23].

CAT’s processing pipeline (see Fig. 2) contains 2 main streams: (i) voxel-based processing for *voxel-based morphometry* (VBM) and (ii) surface-based processing for *surface-based morphometry* (SBM). The former is a prerequisite for the latter, but not the other way round. Both processing streams can be extended to include additional steps for (iii) region-based processing and *region-based morphometry* (RBM).

#### Voxel-based processing

Voxel-based processing steps can be roughly divided into a module for tissue segmentation, followed by a module for spatial registration.

**Figure 3:**
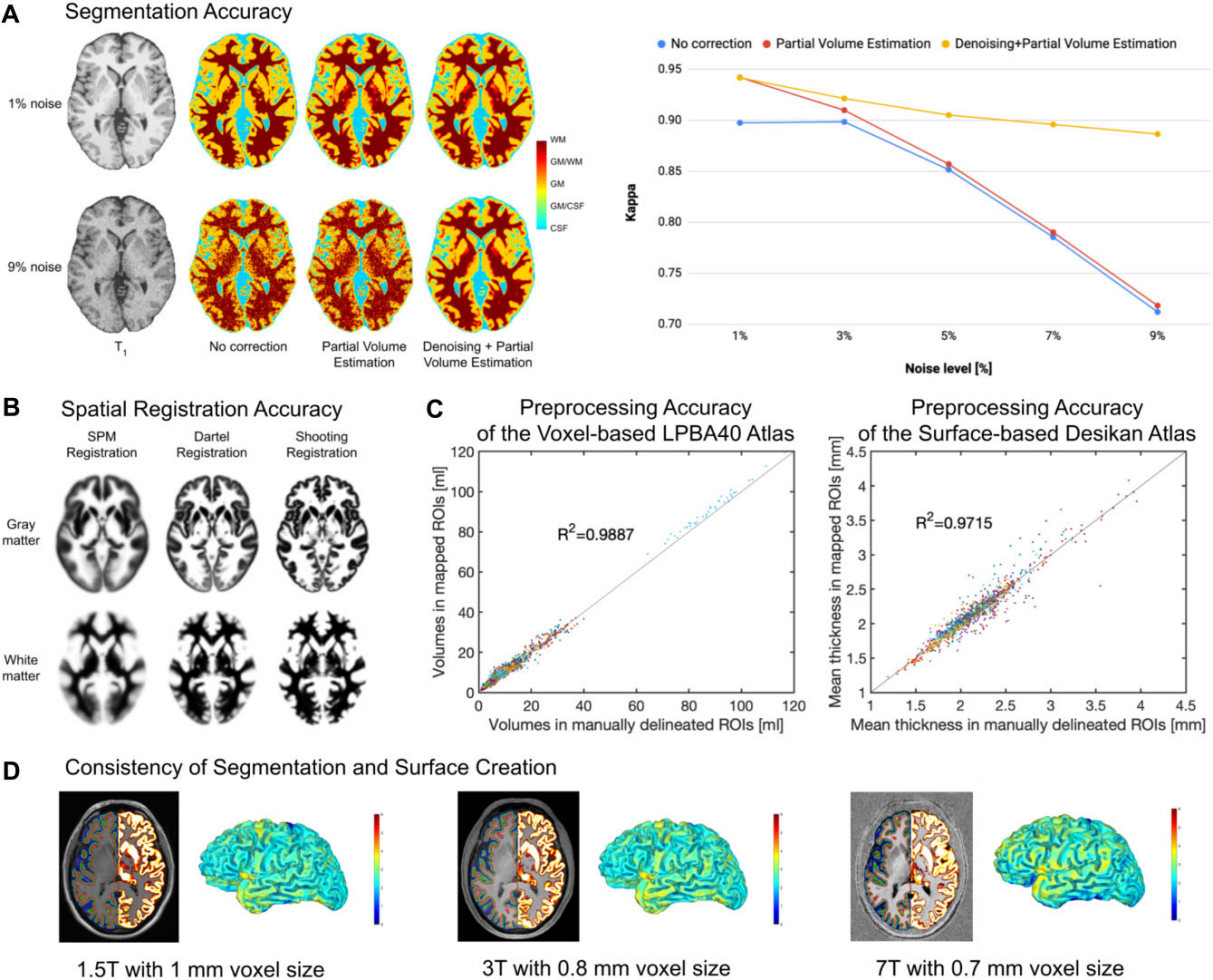
Evaluation of segmentation and registration accuracy. (A) *Segmentation Accuracy:* Most approaches for brain segmentation assume that each voxel belongs to a particular tissue class, such as *gray matter* (GM), *white matter* (WM), or *cerebrospinal fluid* (CSF). However, the spatial resolution of brain images is limited, leading to so-called *partial volume effects* (PVE) in voxels containing a mixture of different tissue types, such as GM/WM and GM/CSF. As PVE approaches are highly susceptible to noise, we combined the PVE model [18] with a spatial adaptive nonlocal means denoising filter [15]. To validate our method, we used a ground-truth image from the BrainWeb [31] database with varying noise levels of 1–9%. The segmentation accuracy for all tissue types (GM, WM, CSF) was determined by calculating a *kappa* coefficient (a kappa coefficient of 1 means that there is perfect correspondence between the segmentation result and the ground truth). *Left panel:* The effect of the PVE model and the denoising filter on the tissue segmentation at the extremes of 1% and 9% noise. *Right panel:* The kappa coefficient over the range of different noise levels. Both panels demonstrate the advantage of combining the PVE model with a spatial adaptive nonlocal means denoising filter, with particularly strong benefits for noisy data. (B) *Registration Accuracy:* To ensure an appropriate overlap of corresponding anatomical regions across brains, high-dimensional nonlinear spatial registration is required. CAT uses a sophisticated shooting approach [24], together with an average template created from the IXI dataset [32]. The figure shows the improved accuracy (i.e., a more detailed average image) when spatially registering 555 brains using the so-called shooting registration and the Dartel registration compared to the SPM standard registration. (C) *Preprocessing Accuracy:* We validated the performance of *region-based morphometry* (RBM) in CAT by comparing measures derived from automatically extracted *regions of interest* (ROI) versus manually labeled ROIs. For the voxel-based analysis, we used 56 structures, manually labeled in 40 brains that provided the basis for the LPBA40 atlas [33]. The gray matter volumes from those manually labeled regions served as the ground truth against which the gray matter volumes calculated using CAT and the LPBA40 atlas were then compared. For the surface-based analysis, we used 34 structures that were manually labeled in 39 brains according to Desikan et al. [34]. The mean cortical thickness from those manually labeled regions served as the ground truth against which the mean cortical thickness calculated using CAT and the Desikan atlas were compared. The diagrams show excellent overlap between manually and automatically labeled regions in both voxel-based (left) and surface-based (right) analyses. (D) *Consistency of Segmentation and Surface Creation:* Data from the same brain were acquired on MRI scanners with different isotropic spatial resolutions and different field strengths: 1.5T MPRAGE with a 1-mm voxel size, 3T MPRAGE with a 0.8-mm voxel size, and 7T MP2RAGE with a 0.7-mm voxel size. *Section Views:* The left hemispheres depict the central (*green*), pial (*blue*), and white matter (*red*) surfaces; the right hemispheres show the gray matter segments. *Rendered Views:* The color bar encodes point-wise cortical thickness projected onto the left hemisphere central surface. Both section views and hemisphere renderings demonstrate the consistency of the outcomes of the segmentation and surface creation procedures across different spatial resolutions and field strengths.

- Tissue Segmentation: The process is initiated by applying a *spatially adaptive nonlocal means* (SANLM) denoising filter [15], followed by SPM’s standard *unified segmentation* [16]. The resulting output serves as a starting point for further optimizations and CAT’s tissue segmentation steps: first, the brain is parcellated into the left and right hemispheres, subcortical areas, ventricles, and cerebellum. In addition, local white matter hyperintensities are detected (to be later accounted for during the spatial registration and the optional surface processing). Second, a local intensity transformation is performed to reduce the effects of higher gray matter intensities in the motor cortex, basal ganglia, and occipital lobe, which are influenced by varying degrees of myelination. Third, an *adaptive maximum a posteriori* (AMAP) segmentation is applied, which does not require any *a priori* information on the tissue probabilities [17]. The AMAP segmentation also includes a *partial volume estimation* [18]. Figure 3A provides information on the accuracy of CAT’s tissue segmentation.
- Spatial Registration: Geodesic Shooting [24] is used to register the individual tissue segments to standardized templates in the ICBM 2009c Nonlinear Asymmetric space (*MNI152NLin2009cAsym* [25]), hereafter referred to as MNI space. While MNI space is also used in many other software packages, enabling cross-study comparisons, users may also choose to use their own templates. Figure 3B provides information on the accuracy of CAT’s spatial registration.

#### Voxel-based morphometry (VBM)

VBM is applied to investigate the volume (or local amount) of a specific tissue compartment [16, 26]—usually gray matter. VBM incorporates different processing steps: (i) tissue segmentation and (ii) spatial registration, as detailed above, and in addition, (iii) adjustments for volume changes due to the registration (modulation) as well as (iv) convolution with a 3-dimensional (3D) Gaussian kernel (spatial smoothing). As a side note, the modulation step results in voxel-wise gray matter volumes that are the same as in native space (i.e., before spatial registration) and not corrected for brain size yet. To remove effects of brain size, users have at least 2 options: (i) calculating the *total intracranial volume* (TIV) and including TIV as a covariate in the statistical model [27] or (ii) selecting “global scaling” (see second-level options in SPM). The latter is recommended if TIV is linked with (i.e., not orthogonal to) the effect of interest (e.g., sex), which can be tested (see “Design orthogonality” in SPM).

#### Surface-based processing

The optional surface-based processing comprises a series of steps that can be roughly divided into a module for surface creation, followed by a module for surface registration.

- Surface Creation: Fig. 3 illustrates the surface creation step in CAT for data obtained on scanners with different field strengths (1.5, 3.0, and 7.0 Tesla). CAT uses a projection-based thickness method [19], which estimates the initial cortical thickness and initial central surface in a combined step, while handling partial volume information, sulcal blurring, and sulcal asymmetries, without explicit sulcus reconstruction. After this initial step, topological defects (i.e., anatomically incorrect connections between gyri or sulci) are repaired using spherical harmonics [20]. The topological correction is followed by a surface refinement, which results in the final central, pial, and white surface meshes. In the last step, the final pial and white matter surfaces are used to refine the initial cortical thickness estimate using the FreeSurfer thickness metric [22, 23]. Alternatively, the final central surface can be used to calculate metrics of cortical folding, as described under “Surface-based morphometry (SBM).”
- Surface Registration: The resulting individual central surfaces are registered to the corresponding hemisphere of the FreeSurfer *FsAverage* template [28]. During this process, the individual central surfaces are spherically inflated with minimal distortions [29], and a one-to-one mapping between the folding patterns of the individual and template spheres is created by a 2-dimensional (2D) version of the DARTEL approach [21, 30]. Figure 3D provides information on the accuracy of CAT’s surface registration.

#### Surface-based morphometry (SBM)

**Figure 4:**
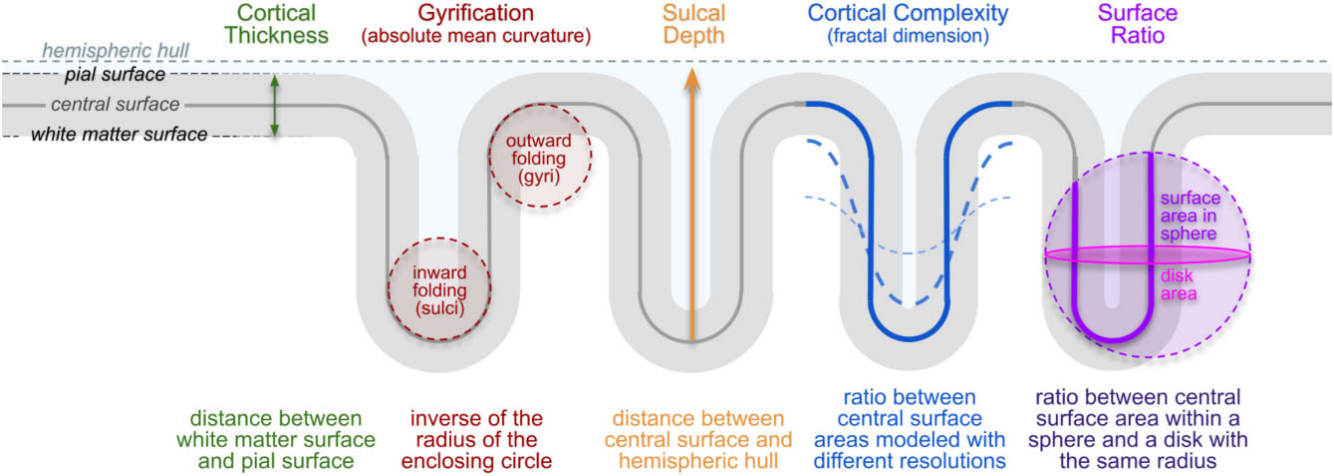
*Cortical Measurements:* Surface-based morphometry is applied to investigate cortical surface features (i.e., cortical thickness and various parameters of cortical folding) at thousands of surface points. *Cortical Thickness*: One of the best-known and most frequently used morphometric measures is cortical thickness, which captures the width of the gray matter ribbon as the distance between its inner boundary (white matter surface) and outer boundary (pial surface). *Cortical Folding*: CAT provides distinct cortical folding measures, derived from the geometry of the central surface: “Gyrification” is calculated via the absolute mean curvature [35] of the central surface. “Sulcal Depth” is calculated as the distance from the central surface to the enclosing hull [36]. “Cortical Complexity” is calculated using the fractal dimension of the central surface area from spherical harmonic reconstructions [37]. Finally, “Surface Ratio” is calculated as the ratio between the area of the central surface contained in a sphere of a defined size and that of a disk with the same radius [38].

SBM can be used to investigate cortical thickness or various parameters of cortical folding. The measurement of “cortical thickness” captures the width of the gray matter ribbon as the distance between its inner and outer boundary at thousands of points (see Fig. 4). To obtain measurements of “cortical folding,” the user has a variety of options in CAT, ranging from *Gyrification* [35] to *Sulcal Depth* [36] to *Cortical Complexity* [37] to the *Surface Ratio* [38], as explained and illustrated in Fig. 4. Similar to VBM, SBM incorporates a series of different steps: (i) surface creation and (ii) surface registration, as detailed above, and (iii) spatial smoothing. As a side note, since the measurements in native space are mapped directly to the template during the spatial registration, no additional modulation (as in VBM) is needed to preserve the individual differences. In contrast to VBM, SBM does not require brain size corrections because cortical thickness and cortical folding are not closely associated with total brain volume (unlike gray matter volume) [39].

#### Region-based processing and morphometry

In addition to voxel- or point-wise analyses via VBM or SBM, CAT provides an option to conduct regional analyses via *region-based morphometry* (RBM). For this purpose, the processing steps under voxel-based processing (surface-based processing, respectively) should be applied and followed by automatically calculating regional measurements. This is achieved by working with regions of interest (ROIs), defined using standardized atlases. The required atlases are provided in CAT (see Supplementary Table S1 and Supplementary Table S2), but users can also work with their own atlases.

- Voxel-based ROIs: The volumetric atlases available in CAT have been defined on brain templates in MNI space and may be mapped to the individual brains by using the spatial registration parameters determined during voxel-based processing. Volumetric measures, such as regional gray matter volume, can then be calculated for each ROI in native space.
- Surface-based ROIs: The surface atlases available in CAT are supplied on the *FsAverage* surface and can be mapped to the individual surfaces by using the spherical registration parameters determined during the surface-based processing. Surface-based measures, such as cortical thickness or cortical folding, are then calculated for each ROI in native space.

### Performance of CAT

CAT allows processing streams to be distributed to multiple processing cores, to reduce processing time. For example, CAT’s analysis of 50 subjects (see “Example application”), leveraging the inbuilt parallel processing capabilities on 4 cores, required 7 hours of processing time when analyzing 1 image per subject (cross-sectional stream) and 18 hours when processing 3 images per subject (longitudinal stream) for the entire sample. Application of all available workflows for a single T1-weighted image takes around 35 minutes, as timed on an iMac with Intel Core i7 with 4 GHz and 32 GB RAM using MATLAB version 2017b, SPM12 version r7771, and CAT12.8 version r1945.

CAT’s performance has been thoroughly tested by evaluating its accuracy, sensitivity, and robustness in comparison to other tools frequently used in the neuroimaging community. For this purpose, we applied CAT and analyzed real data (see “Example application”) as well as simulated data generated from BrainWeb [40]. The evaluation procedures are detailed in Supplementary Note 1 and Supplementary Note 2; the outcomes are presented in Supplementary Fig. S1 and Supplementary Fig. S2. CAT proved to be accurate, sensitive, reliable, and robust, outperforming other common neuroimaging tools.

### Five selected features of CAT

#### Longitudinal processing

Aside from offering a standard pipeline for cross-sectional analyses, CAT has specific longitudinal pipelines that ensure a local comparability both across subjects and across time points within subjects. Compared to the cross-sectional pipeline, these longitudinal pipelines render analysis outcomes more accurate when mapping structural changes over time. The user can choose between 3 different longitudinal pipelines: the first one for analyzing brain plasticity (over days, weeks, months), the second one for analyzing brain development (over months and years), and the third one for brain aging (over months, years, decades). For more details, refer to Supplementary Note 3.

#### Quality control

CAT introduces a retrospective quality control framework for the empirical quantification of essential image parameters, such as noise, intensity inhomogeneities, and image resolution (all of these can be impacted, for example, by motion artifacts). Separate parameter-specific ratings are provided as well as a handy overall rating [41]. Moreover, image outliers can be easily identified, either directly based on the aforementioned indicators of the image quality or by calculating a *z*-score determined by the quality of the image processing as well as by the anatomical characteristics of each brain. For more details, refer to Supplementary Note 4.

#### Mapping onto the cortical surface

CAT allows the user to map voxel-based values (e.g., quantitative, functional, or diffusion parameters) to individual brain surfaces (i.e., pial, central, and/or white matter) for surface-based analyses. The integrated equi-volume model [42] also considers the shift of cytoarchitectonic layers caused by the local folding. Optionally, CAT also allows mapping of voxel values at multiple positions along the surface normal at each node—supporting a layer-specific analysis of ultra-high resolution functional MRI data [43, 44]. For more details, refer to Supplementary Note 5.

#### Threshold-free cluster enhancement (TFCE)

CAT comes with its own threshold-free cluster enhancement (TFCE) toolbox and provides the option to apply TFCE [45] in any statistical *second-level* analysis in SPM, for both voxel-based and surface-based analyses. It can also be employed to analyze *functional MRI* (fMRI) or *diffusion tensor imaging* (DTI) data. A particularly helpful feature of the TFCE toolbox is that it automatically recognizes exchangeability blocks and potential nuisance parameters [46] from an existing statistical design in SPM. For more details, refer to Supplementary Note 4.

#### Visualization

**Figure 5:**
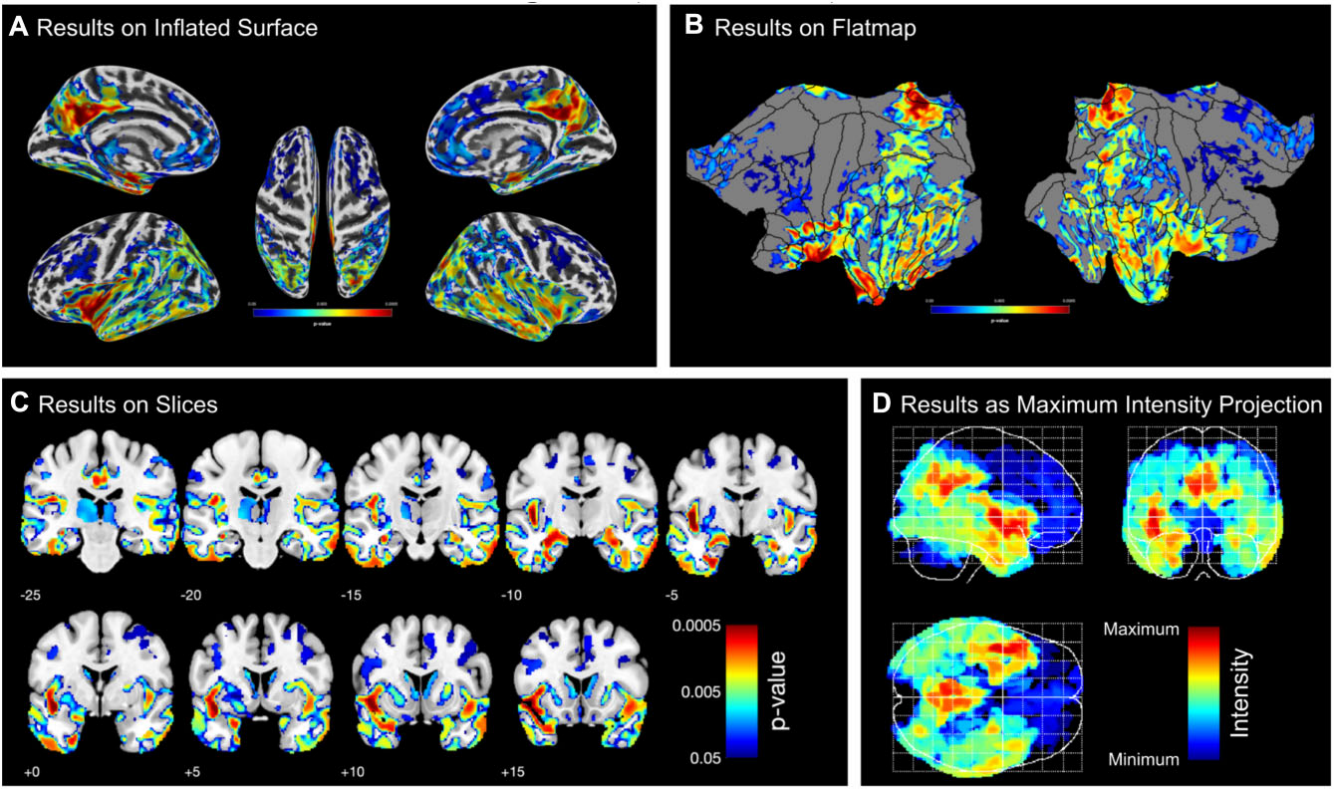
Examples of CAT’s visualization of results. Both surface- and voxel-based data can be presented on surfaces such as (A) the (inflated) *FsAverage* surface or (B) the flatmap of the Connectome Workbench. Volumetric maps can also be displayed as (C) slice overlays on the MNI average brain or (D) a maximum intensity projection (so-called glass brains). All panels show the corrected *P* values from the longitudinal VBM study in our example (see “Example application”).

CAT allows a user to generate graphs and images, which creates a solid basis to explore findings as well as to generate ready-to-publish figures according to prevailing standards. More specifically, it includes 2 distinct sets of tools to visualize results: the first set prepares both voxel- and surface-based data for visualization by providing options for thresholding the default SPM *T*-maps or *F*-maps and for converting statistical parameters (e.g., *T*-maps and *F*-maps into *p*-maps). The second set of tools visualizes the data offering the user ample options to select from different brain templates, views, slices, significance parameters, significance thresholds, color schemes, and so on (see Fig. 5).

### Example application

To demonstrate an application of CAT, we investigated an actual dataset focusing on the effects of Alzheimer’s disease on brain structure. More specifically, we set out to compare 25 patients with Alzheimer’s disease and 25 matched controls. We applied (i) a VBM analysis focusing on voxel-wise gray matter volume, (ii) an RBM analysis focusing on regional gray matter volume (i.e., a voxel-based ROI analysis), (iii) a surface-based analysis focusing on point-wise cortical thickness, and (iv) an RBM analysis focusing on regional cortical thickness (i.e., a surface-based ROI analysis). Given the wealth of literature on Alzheimer’s disease, we expected atrophy in gray matter volume and cortical thickness in patients compared to controls, particularly in regions around the medial temporal lobe and the default mode network [47, 48]. In addition to distinguishing between the 4 morphological measures (i–iv), all analyses were conducted using both cross-sectional and longitudinal streams in CAT. Overall, we expected that longitudinal changes would manifest in similar brain regions to cross-sectional group differences but that cross-sectional effects would be more pronounced than longitudinal effects. The outcomes of this example analysis are presented and discussed in the next section.

## Discussion

### Example application

**Figure 6:**
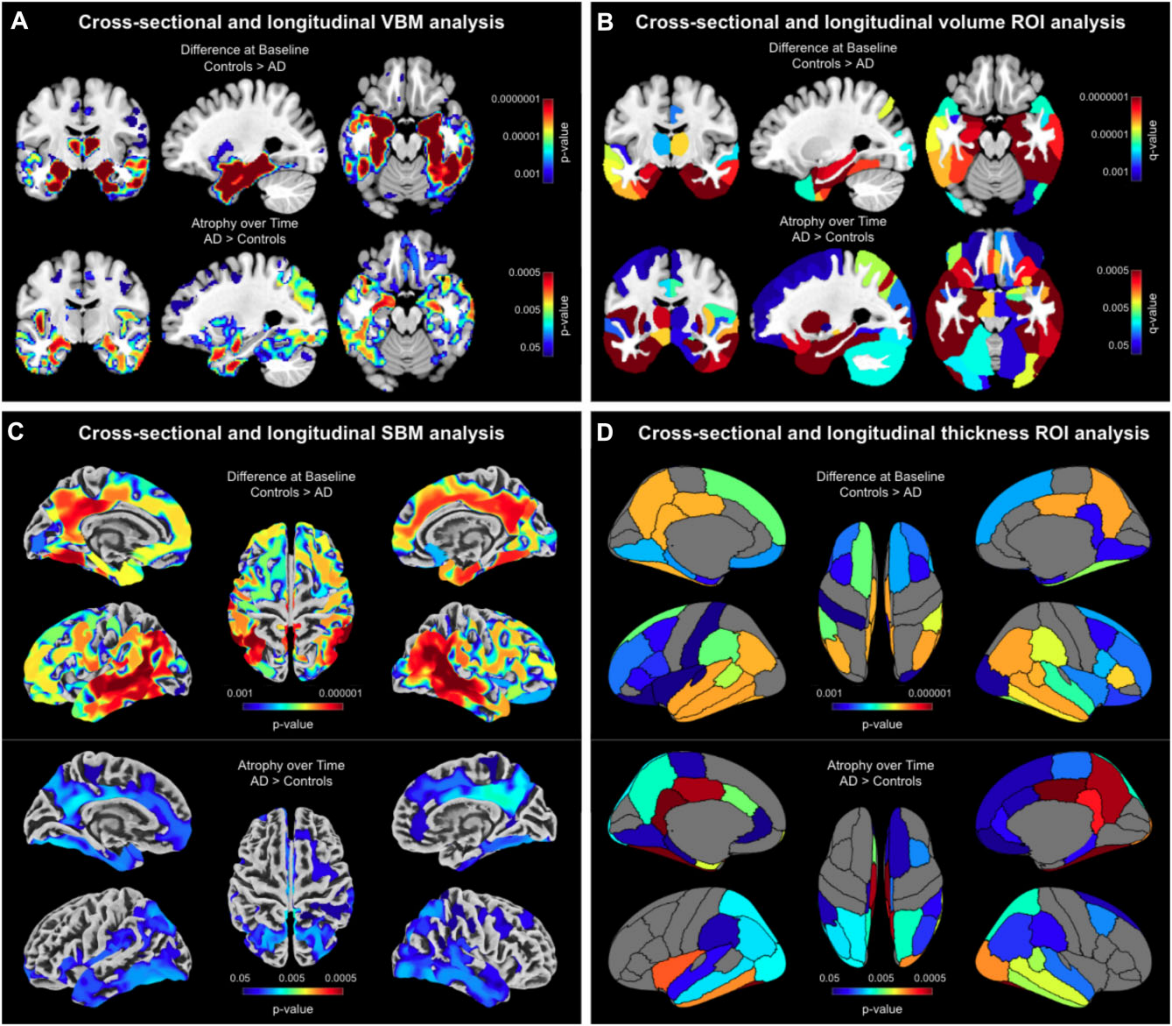
Pronounced atrophy in gray matter and cortical thickness in patients with Alzheimer’s disease compared to healthy control subjects. (A) *Voxel-based morphometry* (VBM) findings: Results were estimated using *threshold-free cluster enhancement* (TFCE), corrected for multiple comparisons by controlling the *family-wise error* (FWE), and thresholded at *P* < 0.001 for cross-sectional data and *P* < 0.05 for longitudinal data. Significant findings were projected onto orthogonal sections intersecting at (x = −27 mm, y = −10 mm, z = −19 mm) of the mean brain created from the entire study sample (*n* = 50). (B) Volumetric *regions of interest* (ROI) findings: ROIs were defined using the Neuromorphometrics atlas. Results were corrected for multiple comparisons by controlling the *false discovery rate* (FDR) and thresholded at *q* < 0.001 for cross-sectional data and *q* < 0.05 for longitudinal data. Significant findings were projected onto the same orthogonal sections as for the VBM findings. (C) *Surface-based morphometry* (SBM) findings: Results were estimated using TFCE, FWE-corrected, and thresholded at *P* < 0.001 for cross-sectional data and *P* < 0.05 for longitudinal data. Significant findings were projected onto the FreeSurfer *FsAverage* surface. (D) Surface ROI findings: ROIs were defined using the DK40 atlas. Results were FDR-corrected and thresholded at *q* < 0.001 for cross-sectional data and *q* < 0.05 for longitudinal data. Significant findings were projected onto the *FsAverage* surface.

As shown in Fig. 6, all 4 cross-sectional streams—investigating voxel-based gray matter volume, regional gray matter volume, point-wise thickness, and regional thickness—revealed widespread group differences between patients with Alzheimer’s disease (AD) and matched controls. Overall, the effects were comparable between cross-sectional and longitudinal streams, but the significant clusters were more pronounced cross-sectionally (note the different thresholds cross-sectionally and longitudinally).

More specifically, using VBM, significantly smaller voxel-wise gray matter volumes were observed in patients with AD compared to controls, particularly in the medial and lateral temporal lobes and within regions of the default mode network (Fig. 6A, top). Similarly, the longitudinal follow-up revealed a significantly stronger gray matter volume loss in patients compared to controls, with effects located in the medial temporal lobe as well as the default mode network (Fig. 6A, bottom). The voxel-based ROI analysis resulted in a significance pattern similar to the VBM study, with particularly pronounced group differences in the temporal lobe that extended into additional brain areas, including those comprising the default mode network (Fig. 6B, top). Again, the longitudinal analysis yielded similar but less pronounced findings than the cross-sectional analysis, although longitudinal effects were stronger than in the VBM analysis (Fig. 6B, bottom).

Using SBM, the point-wise cortical thickness analysis yielded a pattern similar to the VBM analysis with significantly thinner cortices in patients, particularly in the medial and lateral temporal lobe and within regions of the default mode network (Fig. 6C, top). Just as in the VBM analysis, significant clusters were widespread and reached far into adjacent regions. Again, the results from the longitudinal stream were less widespread and significant than the results from the cross-sectional stream (Fig. 6C, bottom). Finally, the surface-based ROI analysis largely replicated the local findings from the SBM analysis (Fig. 6D, top/bottom).

Overall, the results of all analysis streams corroborate prior findings in the Alzheimer’s disease literature, particularly the strong disease effects within the medial temporal lobe and regions of the default mode network [47, 48]. Furthermore, the comparable pattern across measures suggests a considerable consistency between available morphometric options, even if gray matter volume and cortical thickness are biologically different and not perfectly related [49, 50].

### Evaluation of CAT12

As shown in Supplementary Fig. S1 and Supplementary Fig. S2, CAT12 proved to be accurate, sensitive, reliable, and robust, outperforming other common neuroimaging tools. Similar conclusions have been drawn in independent evaluations testing 1 or more software in comparison with CAT12. For example, Guo et al. [51] evaluated the repeatability and reproducibility of brain volume measurements using FreeSurfer, FSL-SIENAX, and SPM and highlighted the reliability of CAT12. Similarly, CAT12 emerged as a robust option when demonstrating that the choice of the processing pipeline influences the location of neuroanatomical brain markers [52]. Last but not least, Khlif et al. [53] compared the outcomes of CAT12’s automated segmentation of the hippocampus with those achieved based on manual tracing and demonstrated that both approaches produced comparable hippocampal volume.

In addition, numerous evaluations suggest that CAT12 performs at least as well as other common neuroimaging tools and, as such, offers a valuable alternative. For example, Tavares et al. [54] conducted a VBM study and concluded that the segmentation pipelines implemented in CAT12 and SPM12 provided results that are highly correlated and that the choice of the pipeline had no impact on the accuracy of any brain volume measure. Along the same lines, but for SBM, Ay et al. [55] reported that CAT12 and FreeSurfer produced equally valid results for parcel-based cortical thickness calculations. de Fátima Machado Dias et al. [56] addressed the issue of reproducibility and observed that cortical thickness measures using CAT12 and FreeSurfer were comparable at the individual level. Moreover, Seiger et al. [57] conducted a study in patients with Alzheimer’s disease and healthy controls, in which CAT12 and FreeSurfer provided consistent cortical thickness estimates and excellent test–retest variability scores. Velázquez et al. [58] supported these findings when comparing CAT12 and FreeSurfer with 3 voxel-based methods in a test–retest analysis and clinical application. Finally, Righart et al. [59] compared volume and surface-based cortical thickness measurements in multiple sclerosis and emphasized CAT12’s consistent performance.

These collective findings from multiple studies support the notion that CAT is a robust and reliable tool for both VBM and SBM analyses, producing results that are comparable to and, in some cases, superior to other established neuroimaging software.

### Conclusion

CAT is suitable for desktop and laptop computers as well as high-performance clusters. It is fully integrated into the SPM environment within MATLAB but also allows process execution directly from the command shell, without having to start SPM. CAT can also run without a MATLAB license by using the stand-alone version or by using Octave instead of MATLAB. In terms of performance, CAT allows for ultra-fast processing and analysis and also is more sensitive in detecting significant effects compared to other common tools used by the neuroimaging community. Moreover, it better handles varying levels of noise and signal inhomogeneities. Furthermore, CAT is easy to integrate with non-SPM software packages and also supports the Brain Imaging Data Structure (BIDS) standards [60]. Therefore, CAT is ideally suited to process not only small datasets (as demonstrated in the example application) but also big datasets, such as samples of the UK Biobank [61] or ENIGMA [62]. Finally, while CAT is currently targeted at structural imaging data, some features (e.g., high-dimensional spatial registration or mapping onto the cortical surface) may also be used for the analysis of functional, diffusion, or quantitative MRI or EEG/MEG data.

## Methods

### Application example

#### Data source

Data for the application example were obtained from the Alzheimer’s Disease Neuroimaging Initiative (ADNI) database [63]. The ADNI (RRID:SCR_003007) was launched in 2003 as a public–private partnership, led by Principal Investigator Michael W. Weiner, MD. The primary goal of ADNI has been to test whether serial MRI, positron emission tomography (PET), other biological markers, and clinical and neuropsychological assessment can be combined to measure the progression of mild cognitive impairment (MCI) and early AD. For up-to-date information, see [64].

#### Sample characteristics

For the purpose of the current study, we compiled a sample of 50 subjects with 3D T1-weighted structural brain images from the ADNI database. Specifically, we randomly selected the first 25 subjects (16 males/9 females) classified as patients with AD (mean age 75.74 ± 8.14 years; mean Minimal Mental Status Examination [MMSE] score: 23.44 ± 2.04) and matched them for sex and age with 25 healthy controls (mean age 76.29 ± 3.90 years; mean MMSE: 28.96 ± 1.24). Informed consent was obtained from all research participants. All subjects had brain scans at baseline (first scan at enrollment) and at 2 follow-up visits, at 1 year and 2 years after the first scan. All brain images were acquired on 1.5 Tesla scanners (Siemens, General Electric, Philips) using a 3D T1-weighted sequence with an in-plane resolution between 0.94 and 1.25 mm and a slice thickness of 1.2 mm.

#### Data processing

All T1-weighted data were processed using CAT12 following the cross-sectional (or longitudinal, respectively) processing stream for VBM, SBM (cortical thickness), and ROI analyses (see Fig. 2) according to the descriptions provided under “Computational morphometry.” For each subject, only their first time point was included in the cross-sectional stream, whereas all 3 time points were included in the longitudinal stream. The processing streams for the VBM analysis resulted in modulated and registered gray matter segments, which were smoothed using a 6-mm Gaussian kernel. The image-processing streams for the SBM analysis resulted in the registered point-wise cortical thickness measures, which were smoothed using a 12-mm Gaussian kernel. The voxel-based ROI analysis used the Neuromorphometrics atlas (RRID:SCR_005656) [65] to calculate the regional gray matter volumes; the surface-based ROI analysis employed the DK40 atlas [34] to calculate regional cortical thickness.

#### Statistical analysis

For each variable of interest—voxel-wise gray matter volume, regional gray matter volume, point-wise cortical thickness, and regional cortical thickness—the dependent measures (e.g., the registered, modulated, and smoothed gray matter segments for voxel-wise gray matter) were entered into the statistical model. For the cross-sectional stream, *group* (patients with AD vs. controls) was defined as the independent variable. For the longitudinal stream, the interaction between *group* and *time* was defined as the independent variable, whereas *subject* was defined as a variable of no interest. For the VBM and the voxel-based ROI analyses, data were corrected for TIV using “global scaling” (because TIV correlated with *group*, the effect of interest). Since cortical thickness does not scale with brain size [39], no corrections for TIV were applied for the SBM and the surface-based ROI analyses. For the cross-sectional analysis, we additionally included age as a nuisance parameter.

For the VBM and SBM analyses, results were corrected for multiple comparisons by applying TFCE [45] and controlling the family-wise error at *P* ≤ 0.001 (cross-sectional) and *P* ≤ 0.05 (longitudinal). For the voxel-based and surface-based ROI analyses, results were corrected for multiple comparisons by controlling the false discovery rate [66] at *q* ≤ 0.001 (cross-sectional) and *q* ≤ 0.05 (longitudinal). All statistical tests were 1-tailed given our *a priori* hypothesis that patients with AD have less gray matter at baseline and a larger loss of gray matter over time.

The outcomes of the VBM and voxel-based ROI analyses were overlaid onto orthogonal sections of the average brain that was created from the spatially registered T1-weighted images of the study sample (*n* = 50); the outcomes of the SBM and surface-based ROI analyses were projected onto the *FsAverage* surface.

## Source Code Availability and Requirements

Project name: Computational Anatomy Toolbox

Project homepage: [9, 69]

Software documentation: [14]

Operating system(s): Platform independent (MacOS, Linux, Windows)

Programming language: MATLAB, C

Other requirements: MATLAB (7.4 or newer)

License: GPL 2.0

RRID:SCR_019184

## Supplementary Material

giae049_GIGA-D-24-00078_Original_Submission

giae049_GIGA-D-24-00078_Revision_1

giae049_GIGA-D-24-00078_Revision_2

giae049_GIGA-D-24-00078_Revision_3

giae049_Response_to_Reviewer_Comments_Original_Submission

giae049_Response_to_Reviewer_Comments_Revision_1

giae049_Response_to_Reviewer_Comments_Revision_2

giae049_Reviewer_1_Report_Original_Submission Chris Armit -- 3/21/2024 Reviewed

giae049_Reviewer_2_Report_Original_Submission Chris Foulon -- 4/10/2024 Reviewed

giae049_Reviewer_3_Report_Original_Submission Cyril Pernet, PhD -- 4/28/2024 Reviewed

giae049_Supplemental_File

## Data Availability

MRI data are available after obtaining approval to access ADNI data at [63]. The BrainWeb data are available at [40]. Snapshots of our code and other data further supporting this work are openly available in the *GigaScience* repository, GigaDB [68].

## Additional Files

**Supplementary Note 1**. Comparison with other tools.

**Supplementary Note 2**. Evaluation with simulated data.

**Supplementary Note 3**. Longitudinal processing.

**Supplementary Note 4**. Quality control.

**Supplementary Note 5**. Mapping onto the cortical surface.

**Supplementary Note 6**. Threshold-free cluster enhancement (TFCE).

**Supplementary Note 7**. Customized methods for clinical data.

**Supplementary Fig. S1**. Comparisons between CAT12 and other common tools. Here we compared the baseline gray matter images of 25 patients with Alzheimer’s disease and 25 matched controls. (a) VBM analyses of voxel-wise gray matter volume using FSL-FAST6 (top), SPM12-Shooting (middle), and CAT12 (bottom). (b) SBM analyses of point-wise cortical thickness using CIVET2.1 (top), Freesurfer7.2 (middle), and CAT12 (bottom). (c, d) Sensitivity of VBM and SBM analyses. The effect sizes (Cohen’s *d*) are shown on the x-axis; their frequency is shown on the y-axis (occurrence is normalized to 1 to facilitate comparisons between histograms). For both VBM and SBM, CAT12 demonstrates a larger sensitivity in detecting structural differences. This is reflected in the more extended significance clusters and lower *P* values (panels a and b) as well as larger effect sizes (panels c and d).

**Supplementary Fig. S2**. Evaluation of CAT12 and other common tools using Brainweb data. Higher kappa values correspond to a better overlap, larger reliability, and increased robustness. (a) Overlap between ground truth and segmentation outputs for different noise levels. CAT12 is similar to FSL-FAST6 at lower noise levels but clearly outperforms both SPM12 and FSL-FAST6 at higher noise levels. The latter is due to the implemented denoising step (see also Fig. 3A for the effect of denoising). (b) Overlap between ground truth and segmentation outputs for different signal inhomogeneities. CAT12 is extremely robust across the entire range of intensity nonuniformity; it outperforms both SPM12 and FSL-FAST6.

**Supplementary Fig. S3**. Comparison between CAT12’s cross-sectional and longitudinal pipelines. Here we compared the longitudinal gray matter images of 25 patients with Alzheimer’s disease and 25 matched controls. Voxel-based morphometry (VBM) results are shown on the left and surface-based morphometry (SBM) results on the right. For both VBM and SBM, the longitudinal preprocessing leads to an increased sensitivity compared to cross-sectional processing, which is evident as larger clusters and lower *P* values (panels a and b) as well as larger effect sizes (panels c and d). The effect sizes are captured as Cohen’s *d* on the x-axis with the frequency of its occurrence normalized to a total sum of 1 (to ease comparisons between histograms) on the y-axis.

**Supplementary Fig. S4**. CAT12’s longitudinal processing workflows to examine (a) neuroplasticity, (b) aging, and (c) neurodevelopment. The first step in all 3 workflows is the creation of a high-quality average image over all time points. For this purpose, CAT12 realigns the images from all time points for each participant using inverse-consistent (or symmetric) rigid-body registrations and intrasubject bias field correction. While this is sufficient to create the required average image for the neuroplasticity and aging workflows, the neurodevelopmental workflow requires nonlinear registrations in addition. In either case, the resulting average image is segmented using CAT12’s regular processing workflow to create a subject-specific tissue probability map (TPM). This TPM is used to enhance the time point–specific processing to create the final segmentations. The final tissue segments are then registered to MNI space to obtain a voxel comparability across time points and subjects, which differs between all 3 workflows. In the neuroplasticity workflow, an average of the time point–specific registrations is created to transform the tissue segments of all time points to MNI space. The aging workflow does the same in principle but adds additional (very smooth) deformations between the individual images across time points to account for inevitable age-related changes over time (e.g., enlargements of the ventricles). In contrast, the neurodevelopmental workflow needs to account for major changes, such as overall head and brain growth, which requires independent nonlinear registrations to MNI space of all images across time points (which are obtained using the default cross-sectional registration model).

**Supplementary Fig. S5**. Subject-specific quality control. Individual quality ratings for each scan are helpful for determining potential problems and issues for the use of single scans. The “Image Quality Ratings” (top) employ measures of noise, bias, and image resolution to generate a summary grade for each image [41]. A “CAT Processing Report” (left) is automatically saved for each image after the processing workflow is completed; it provides information on image quality measures and the overall grade, in addition to visualizations, which allow for an easy assessment of the quality of the skull stripping, tissue segmentation, and surface mapping. Moreover, a “Longitudinal Report” (right) is automatically saved when any of the longitudinal pipelines have been used (see Supplementary Note 3). This longitudinal report—considering all images of 1 brain across all time points—provides the same information as the standard cross-sectional report but focuses on the assessment of differences between the individual time points.

**Supplementary Fig. S6**. Group-specific quality control. In addition to the subject-specific quality control, larger studies in particular might benefit from scrutinizing those images that are either low in their individual quality ratings and/or different from the other images, suggesting anatomic anomalies, imperfect processing, or other issues that might hamper the subsequent statistical analysis. The “Group Boxplot” (left) allows one to compare any image based on their similarity to the mean and reflects the homogeneity of the sample, by calculating the average *z*-score of all spatially registered images (or surface parameter files). Lower average *z*-score values indicate that the data points are more similar to the mean. Outliers (i.e., images with high *z*-score values) indicate either a potential problem (with the image per se or with the outcomes of the image processing) or simply a variation in the neuroanatomy (e.g., enlarged ventricles). Such outliers should be checked carefully. An additional “IQR × Mean Z-Score Window” (right) compares the average *z*-scores with the weighted image quality rating (IQR) for each subject and allows a combined view of sample homogeneity and overall image quality.

**Supplementary Fig. S7**. Volume mapping. CAT12 offers multiple ways to map voxel values onto the surface. The default mapping extracts voxel values at multiple positions along a surface normal between the white matter surface and the pial surface. The exact location of these positions along the normal is determined by an equi-volumetric model [42], which reflects the shift of cortical layers caused by local folding. However, voxel values can also be extracted at a specific user-defined displacement (in mm) from any given surface location.

**Supplementary Table S1**. Voxel-based ROI atlases available in CAT12 (as of October 2023).

**Supplementary Table S2**. Surface-based ROI atlases available in CAT12 (as of October 2023).

## Abbreviations

AD: Alzheimer’s disease; AMAP: adaptive maximum a posteriori; BIDS: Brain Imaging Data Structure; CAT: Computational Anatomy Toolbox; CSF: cerebrospinal fluid; DTI: diffusion tensor imaging; EEG: electroencephalography; FDR: false discovery rate; fMRI: functional magnetic resonance imaging; FWE: family-wise error; FWHM: full width at half maximum; GM: gray matter; IQR: image quality rating; MEG: magnetoencephalography; MMSE: Minimal Mental Status Examination; MNI: Montreal Neurological Institute; MPRAGE: Magnetization Prepared Rapid Acquisition Gradient Echo; MP2RAGE: Magnetization Prepared 2 Rapid Acquisition Gradient Echoes; MRI: magnetic resonance imaging; PBT: projection-based thickness; PVE: partial volume estimation; RBM: region-based morphometry; ROI: region of interest; SANLM: spatially adaptive nonlocal means; SBM: surface-based morphometry; SLC: stroke lesion correction; SPM: statistical parametric mapping; TFCE: threshold-free cluster enhancement; TIV: total intracranial volume; TPM: tissue probability map; VBM: voxel-based morphometry; WM: white matter; WMH: white matter hyperintensity; WMHC: white matter hyperintensity correction.
