# Supplementary material for: CAT: a computational anatomy toolbox for the analysis of structural MRI data
Source: Gigascience. 2024 Aug 5;13:giae049. doi: 10.1093/gigascience/giae049 (PMC11299546; doi:10.1093/gigascience/giae049)
Supplement: giae049_Supplemental_File [file giae049_supplemental_file.pdf]

Supplemental Material

CAT – A Computational Anatomy Toolbox for the Analysis of Structural MRI Data

Christian Gaser[a,b,c*], Robert Dahnke[a,b,c], Paul M Thompson[d], Florian Kurth[e,f+], Eileen Luders[e,g,h,i+], and the **Alzheimer's Disease Neuroimaging Initiative[1]**

[a]Department of Psychiatry and Psychotherapy, Jena University Hospital, Jena, Germany

[b]Department of Neurology, Jena University Hospital, Jena, Germany

[c]German Center for Mental Health (DZPG)

[d]Imaging Genetics Center, Stevens Neuroimaging & Informatics Institute, Keck School of Medicine, University of Southern California, Los Angeles, CA, USA

[e]School of Psychology, University of Auckland, Auckland, New Zealand

[f]Departments of Neuroradiology and Radiology, Jena University Hospital, Jena, Germany

[g]Department of Women's and Children's Health, Uppsala University, Uppsala, Sweden

[h]Swedish Collegium for Advanced Study (SCAS), Uppsala, Sweden

[i]Laboratory of Neuro Imaging, School of Medicine, University of Southern California, Los Angeles, CA, USA

[1] Data used in preparation of this article were obtained from the Alzheimer's Disease Neuroimaging Initiative (ADNI) database (adni.loni.usc.edu). As such, the investigators within the ADNI contributed to the design and implementation of ADNI and/or provided data but most did not participate in analysis or writing of this report. A complete listing of ADNI investigators can be found at: http://adni.loni.usc.edu/wp-content/uploads/how_to_apply/ADNI_Acknowledgement_List.pdf

[+]*Shared last authorship*

Supplemental Notes

Supplemental Note 1: Comparison with other tools

We evaluated the performance of CAT12 by comparing it to other tools commonly used in the neuroimaging community. More specifically, we assessed the accuracy and sensitivity using CAT12, SPM12, FSL-FAST6, Freesurfer6 and CIVET 2.1 in detecting subtle alterations in brain structure that are critical for early diagnosis and monitoring of Alzheimer's disease. Note, the primary aim of our comparison is to provide insights into the tool's performances; revealing aberrations associated with Alzheimer's disease is only a secondary aim of this paper. To conduct the comparisons we used the same baseline data of our example application (25 patients with Alzheimer's disease and 25 matched controls), as described in the main article. The analyses focussed on (1) voxel-wise gray matter volume and (2) point-wise cortical thickness. Analyses pertaining to (1) were conducted using voxel-based morphometry (VBM) while processing the data with (1a) SPM version 12 [1] as well as with (1b) FSL-FAST version 6 [2]. Analyses pertaining to (2) were conducted using surface-based morphometry (SBM) while processing the data with (2a) Freesurfer version 7.2 [3] as well as with (2b) Civet version 2.1 [4].

Data Processing for VBM data

1a – SPM12: We applied the Unified Segmentation [5] in SPM12 with default settings to extract rigidly registered gray and white matter segments. These individual segments provided the basis to create a mean segment using the Shooting toolbox [6] in SPM12. This mean segment functions as an initial template and is warped to each of the individual segments, which is followed by calculating the resulting deformations, applying the inverses of the deformations to the individual images, and re-calculating the template (aka the mean segment). This process is repeated several times. The results are spatially registered segments which will be adjusted for volume changes introduced by the registration (modulation) and convoluted with a Gaussian kernel of FWHM 6mm (smoothing).

1b – FSL-FAST6: We applied the FSLVBM script from FSL6 to process the data [7]. The default there is using BET to skull-strip the data. However, the achieved output was of poor quality, which is why we used the aforementioned SPM12 segments (in native space) to skull-strip the data. The skull-stripped data were then processed using the FSLVBM script and smoothed with a 6mm Gaussian kernel, as described above.

Data Processing for SBM data

2a – Freesurfer7.2 The data were processed using the recon-all script for Freesurfer7.2 [3] with default settings. For a better comparison between tools, the resulting cortical thickness measures were resampled and smoothed (FWHM 12 mm) using CAT12.

2b – CIVET2.1: The data were uploaded to CBRAIN (RRID:SCR_005513) [8] and processed with the CIVET2.1 pipeline using default settings. Again, the cortical thickness measures were resampled and smoothed (FWHM 12 mm) using CAT to allow for a better comparison between tools.

Statistical Analysis

For details on the statistical model (e.g., dependent variables, independent variables, and variables of no interest), refer to the Methods section in the main document. All results were

corrected for multiple comparisons by applying TFCE [9] and controlling the family-wise error at $p<0.001$. All statistical tests were one-tailed given our a priori hypothesis that AD patients present with less gray matter at baseline and a larger loss of gray matter over time. In addition, we calculated the effect sizes to allow for a direct comparison across tools with respect to their sensitivity in detecting significant differences between AD patients and controls.

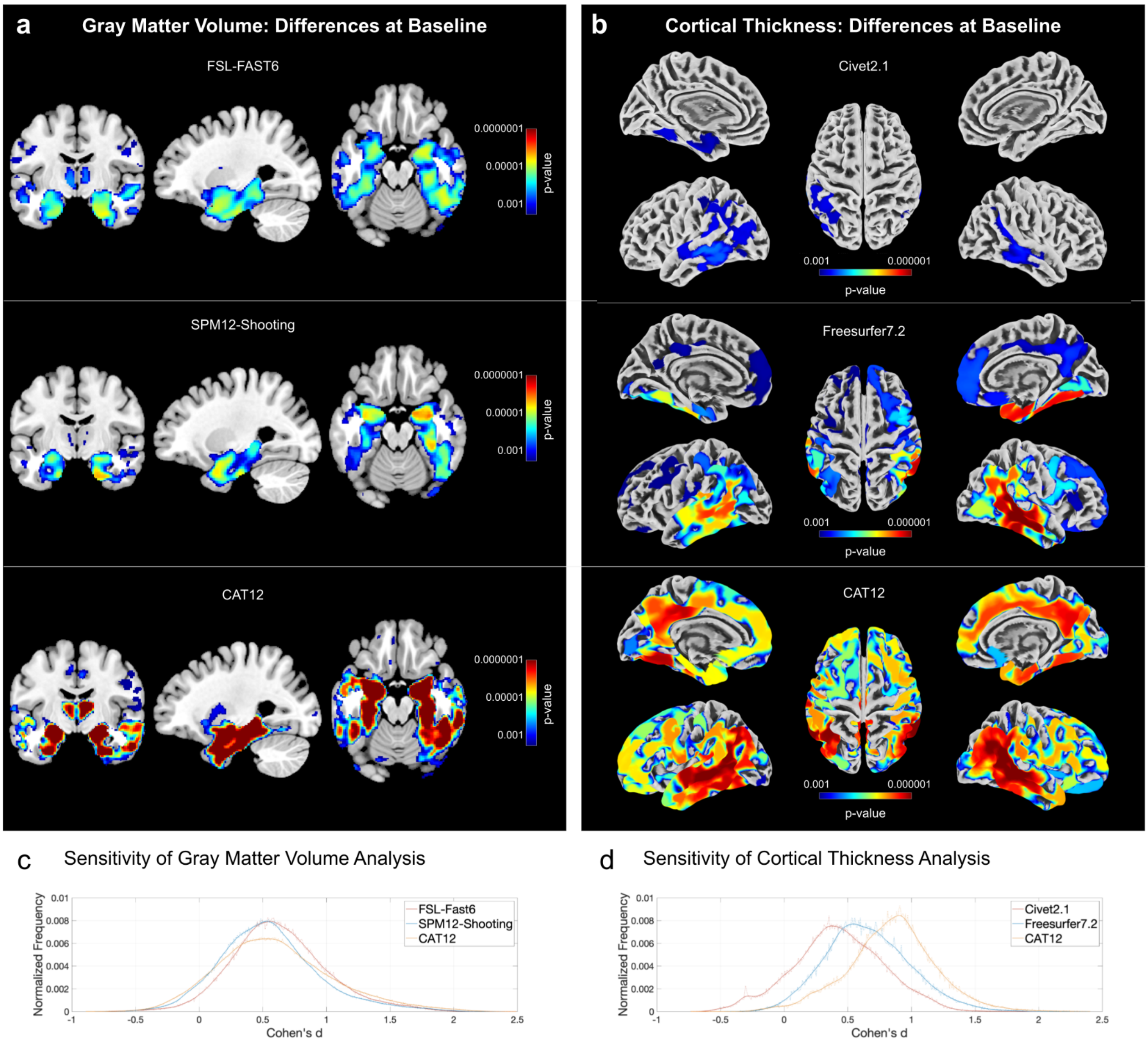

Supplemental Figure 1: Comparisons between CAT12 and other common tools. Here we compared the baseline gray matter images of 25 patients with Alzheimer's disease and 25 matched controls. *Panel a:* VBM analyses of voxel-wise gray matter volume using FSL-FAST6 (*top*), SPM12-Shooting (*middle*), and CAT12 (*bottom*). *Panel b:* SBM analyses of point-wise cortical thickness using CIVET2.1 (*top*), Freesurfer7.2 (*middle*), and CAT12 (*bottom*). Panels c and d: Sensitivity of VBM and SBM analyses. The effect sizes (Cohen's *d*) are shown on the *x*-axis; their frequency is shown on the *y*-axis (occurrence is normalized to one to facilitate comparisons between histograms). For both VBM and SBM, CAT12 demonstrates a larger sensitivity in detecting structural differences. This is reflected in the more extended significance clusters and lower *p*-values (*panels a and b*) as well as larger effect sizes (*panels c and d*).

Supplemental Note 2: Evaluation with simulated data

To comprehensively evaluate the performance of CAT12 in comparison with other neuroimaging tools (SPM12 and FSL-FAST6), we conducted evaluations using simulated data generated from BrainWeb [10]. More specifically, we compared the output of CAT12, SPM12, and FSL-FAST6 to ground truth data represented by a brain phantom. As the phantom contains known variations in noise levels and signal inhomogeneities, it aids in objectively assessing the accuracy and robustness of CAT12 and the other tools in dealing with different sources of variation. To measure the agreement between the ground truth and the results of CAT12, SPM12, and FSL-FAST6, we calculated the kappa coefficient.

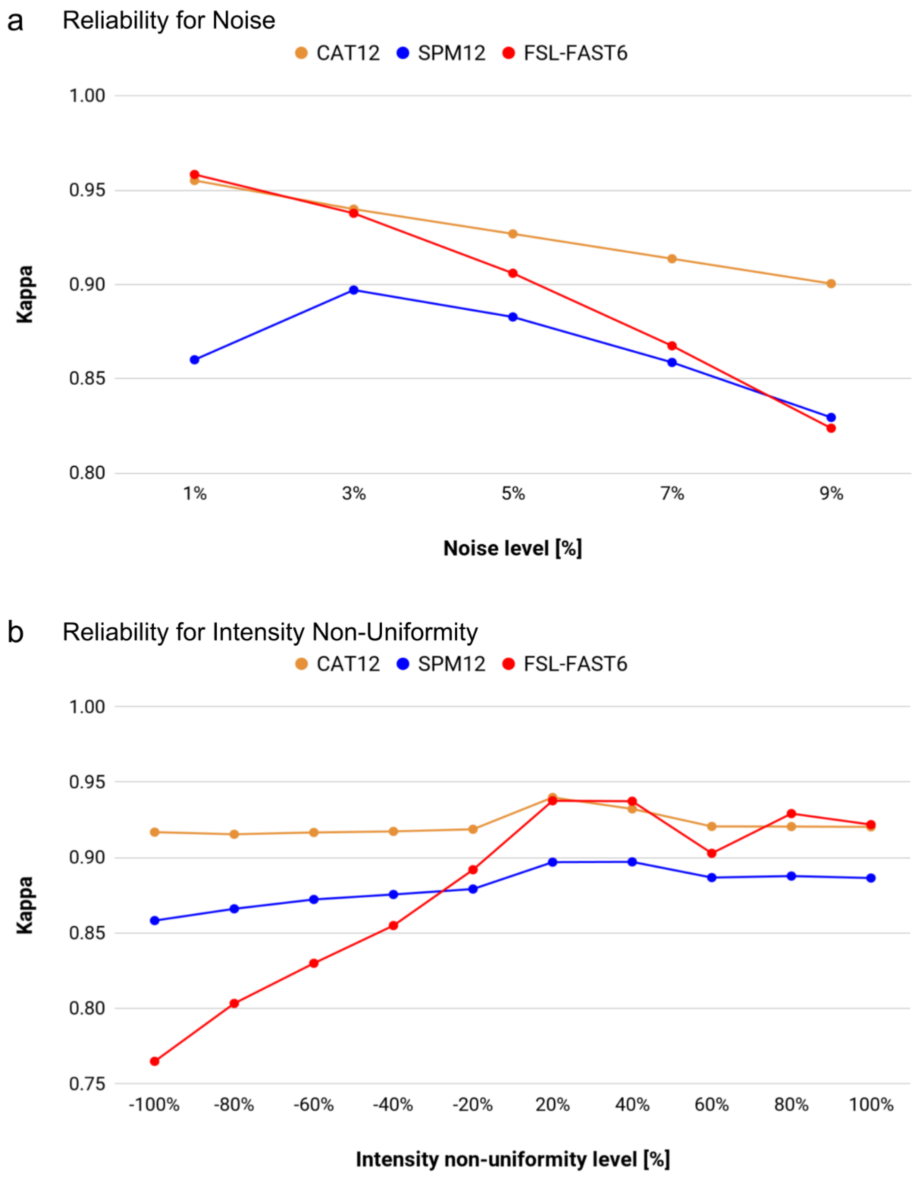

Supplemental Figure 2: Evaluation of CAT12 and other common tools using Brainweb data. Higher *kappa* values correspond to a better overlap, larger reliability, and increased robustness. *Panel a:* Overlap between ground truth and segmentation outputs for different noise levels. CAT12 is similar to FSL-FAST6 at lower noise levels but clearly outperforms both SPM12 and FSL-FAST6 at higher noise levels. The latter is due to the implemented denoising step (see also Figure 3a for the effect of denoising). *Panel b:* Overlap between ground truth and segmentation outputs for different signal inhomogeneities. CAT12 is extremely robust across the entire range of intensity non-uniformity; it outperforms both SPM12 and FSL-FAST6.

Supplemental Note 3: Longitudinal Processing

The majority of morphometric studies are based on cross-sectional data in which one image is acquired for each subject. Nevertheless, the mapping of structural changes over time requires specific longitudinal designs that consider additional time points (and thus images) for each subject. In theory, all images could be processed using the standard cross-sectional processing workflow. In practice, however, longitudinal data strongly benefit from workflows specifically tailored towards longitudinal analyses, where MR-based noise and inhomogeneities are further reduced and where spatial correspondences are ensured, the latter not only across subjects but also across time points within subjects [11–13]. As a consequence, analyses become more sensitive, as shown in **Supplemental Figure 3**.

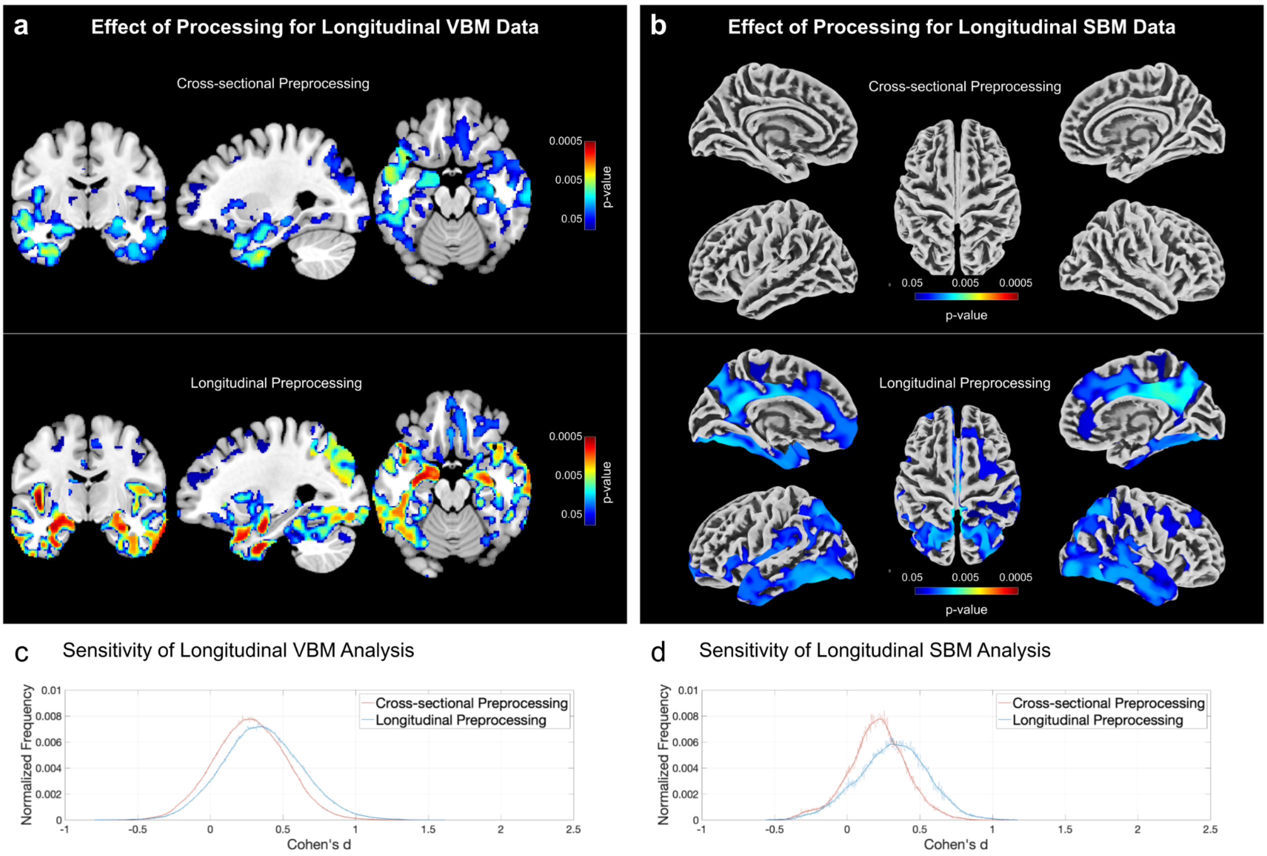

Supplemental Figure 3: Comparison between CAT12's cross-sectional and longitudinal pipelines. Here we compared the longitudinal gray matter images of 25 patients with Alzheimer's disease and 25 matched controls. *Voxel-based morphometry* (VBM) results are shown on the left and *surface-based morphometry* (SBM) results on the right. For both VBM and SBM the longitudinal preprocessing leads to an increased sensitivity compared to cross-sectional processing, which is evident as larger clusters and lower p-values (panels a and b) as well as larger effect sizes (panels c and d). The effect sizes are captured as Cohen's d on the x-axis with the frequency of its occurrence normalized to a total sum of one (to ease comparisons between histograms) on the y-axis.

CAT12 offers three optimized processing pipelines for longitudinal studies: One for neuroplasticity, one for aging, and one for neurodevelopmental studies. Studies in the framework of neuroplasticity are confined to short time-frames of weeks to months, and even days [14,15]. In contrast, studies in the framework of aging and neurodevelopment cover longer time frames of years and, sometimes, even decades. For such extended study durations, it is

particularly important to model systematic changes of the brain over time to maintain a voxel- or point-wise comparability across time points. Studies in the framework of neurodevelopment require additional considerations of increasing brain and head sizes. A detailed description of all three longitudinal processing workflows is provided in **Supplemental Figure 4**.

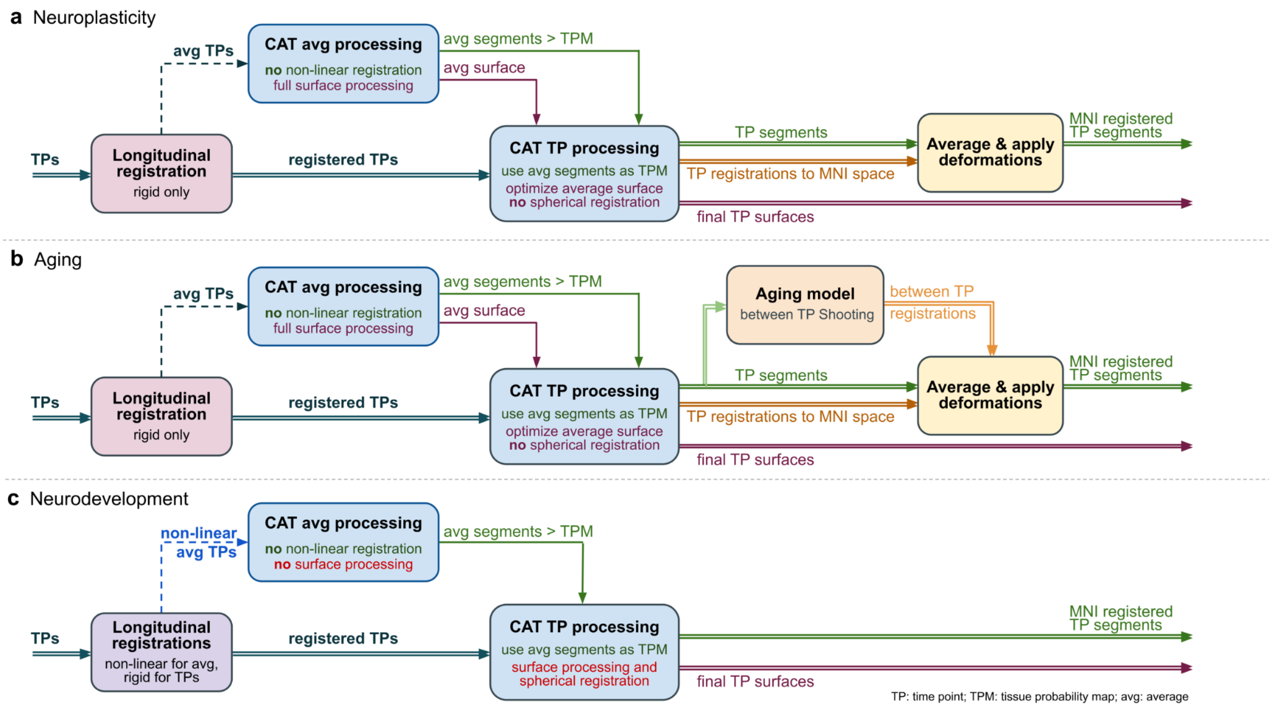

Supplemental Figure 4: CAT12's longitudinal processing workflows to examine (a) neuroplasticity, (b) aging, and (c) neurodevelopment. The first step in all three workflows is the creation of a high-quality average image over all time points. For this purpose, CAT12 realigns the images from all time points for each participant using inverse-consistent (or symmetric) rigid-body registrations and intra-subject bias field correction. While this is sufficient to create the required average image for the neuroplasticity and aging workflows, the neurodevelopmental workflow requires non-linear registrations in addition. In either case, the resulting average image is segmented using CAT12's regular processing workflow to create a subject-specific *tissue probability map* (TPM). This TPM is used to enhance the time point-specific processing to create the final segmentations. The final tissue segments are then registered to MNI space to obtain a voxel-comparability across time points and subjects, which differs between all three workflows. In the neuroplasticity workflow, an average of the time point-specific registrations is created to transform the tissue segments of all time points to MNI space. The aging workflow does the same in principle but adds additional (very smooth) deformations between the individual images across time points to account for inevitable age-related changes over time (e.g., enlargements of the ventricles). In contrast, the neurodevelopmental workflow needs to account for major changes, such as overall head and brain growth, which requires independent non-linear registrations to MNI space of all images across time points (which are obtained using the default cross-sectional registration model).

Supplemental Note 4. Quality Control

Processing of MRI data strongly depends on the quality of the input data. Multi-center studies and data sharing projects, in particular, need to take into account varying image properties due to different scanners, sequences and protocols. However, even scans acquired on a single scanner and using the same scanning protocol may vary due to motion or other miscellaneous artifacts. CAT12 provides options to perform quality checks, both on the subject level and on the group level. More specifically, on the subject level, CAT12 introduces a novel retrospective

quality control framework for the quantification of quality differences between different scans obtained on a single scanner or across different scanners. The quality control allows for the evaluation of essential image parameters (i.e., noise, intensity inhomogeneities, and image resolution) and is automatically performed for each brain when running CAT12's image processing workflow (see **Supplemental Figure 5**). On the group-level, CAT12 provides options to check and visualize the homogeneity of the entire study sample, thus allowing the user to identify any outliers (see **Supplemental Figure 6**).

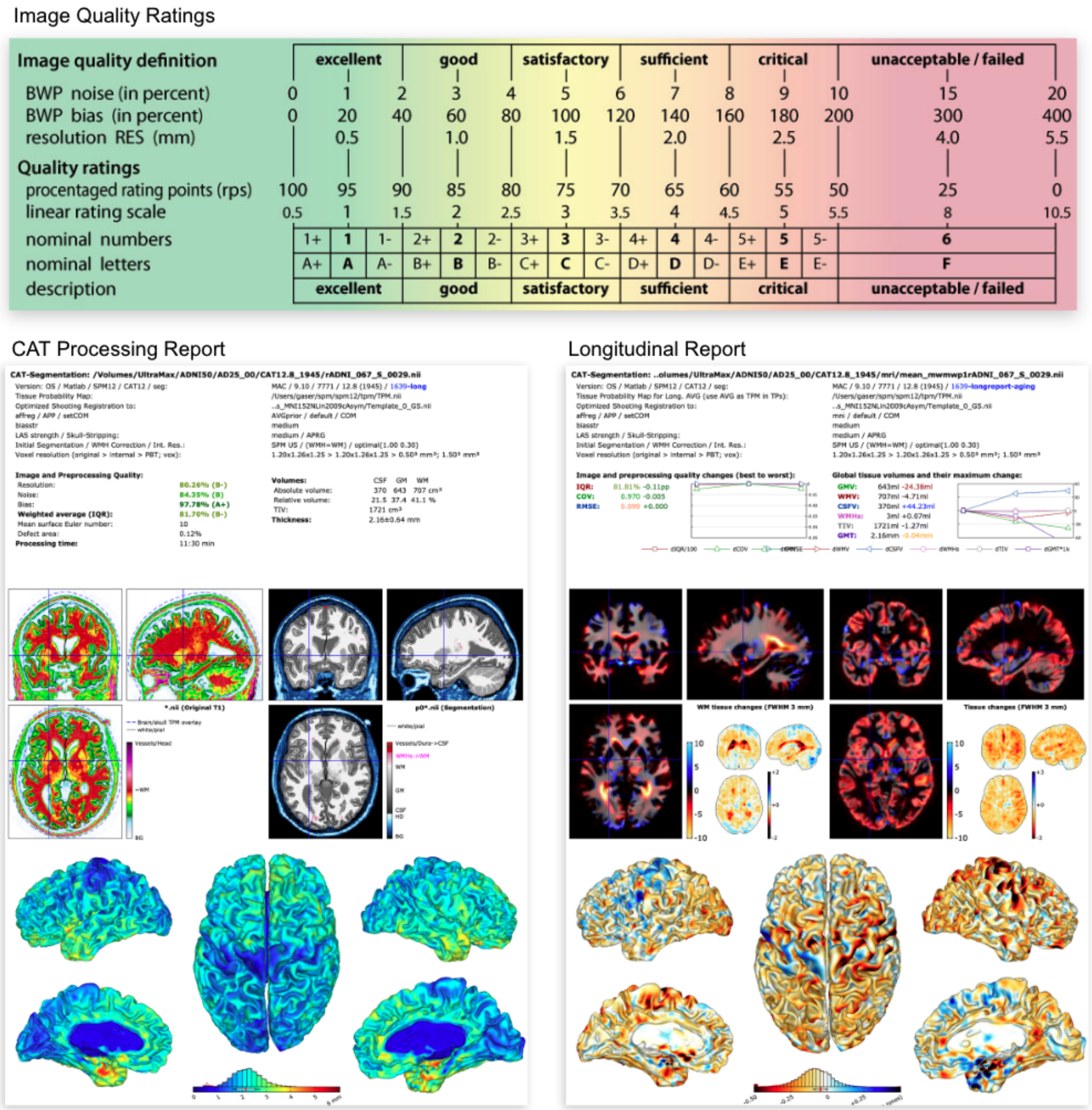

Supplemental Figure 5: Subject-specific quality control. Individual quality ratings for each scan are helpful for determining potential problems and issues for the use of single scans. The 'Image Quality Ratings' (top) employ measures of noise, bias, and image resolution to generate a summary grade for each image [16]. A 'CAT Processing Report' (*left*) is automatically saved for each image after the processing workflow is completed; it provides information on image quality measures and the overall grade, in addition to visualizations which allow for an easy assessment of the quality of the skull stripping, tissue segmentation, and surface mapping. Moreover, a 'Longitudinal Report' (*right*) is automatically saved when any of the longitudinal pipelines have been used (see **Supplemental Note** 3). This longitudinal report – considering all images of one brain across all time points – provides the same information as the standard cross-sectional report but focuses on the assessment of differences between the individual time points.

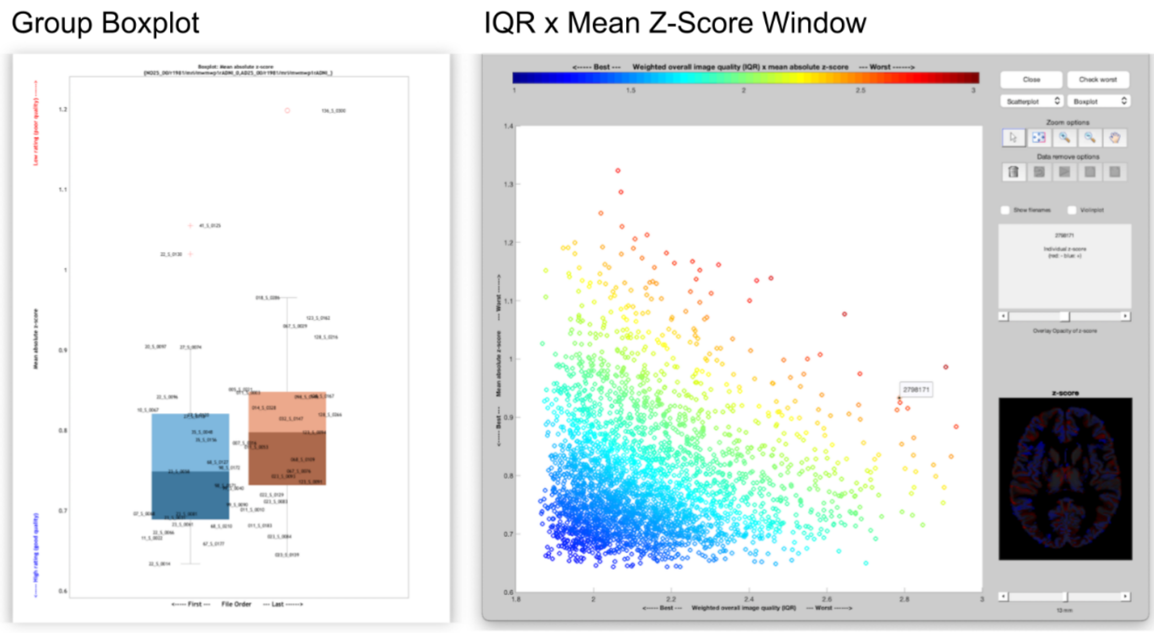

Supplemental Figure 6: Group-specific quality control. In addition to the subject-specific quality control, larger studies in particular might benefit from scrutinizing those images that are either low in their individual quality ratings and/or different from the other images, suggesting anatomic anomalies, imperfect processing, or other issues that might hamper the subsequent statistical analysis. The 'Group Boxplot' (left) allows one to compare any image based on their similarity to the mean and reflects the homogeneity of the sample, by calculating the average Z-score of all spatially registered images (or surface parameter files). Lower average Z-score values indicate that the data points are more similar to the mean. Outliers (i.e., images with high Z-score values) indicate either a potential problem (with the image per se or with the outcomes of the image processing), or simply a variation in the neuroanatomy (e.g., enlarged ventricles). Such outliers should be checked carefully. An additional 'IQR x Mean Z-Score Window' (right) compares the average Z-scores with the weighted image quality rating (IQR) for each subject and allows a combined view of sample homogeneity and overall image quality.

Supplemental Note 5. Mapping onto the Cortical Surface

Surface-based analyses offer some advantages over voxel-based approaches, such as better inter-subject registration and surface-based smoothing, which may result in a larger statistical power and improved accuracy [17,18]. CAT12 provides a range of options to map voxel-based values (e.g., functional, quantitative or diffusion parameters) to individual brain surfaces for a subsequent surface-based analysis. For this purpose, voxel-based values are extracted at multiple positions along the surface normal at each node of the surface (see **Supplemental Figure 7**). The exact positions along the surface normal are determined by an equi-volume model [19], which reflects the normal shift of cytoarchitectonic layers caused by the local folding. In addition to default settings, users can specify both the number and location of those positions along the surface normal. The extracted values along the surface normal are then summarized as one value per node. The default here is to summarize values by using the absolute maximum value. However, other options than using the absolute maximum exist, such as using the minimum, mean, or weighted mean value. Alternatively, users may choose to map voxel values at a specified distance (in mm) from the surface or even at multiple positions along the surface normal. The latter is useful, for example, when conducting a layer-specific analysis of ultra-high resolution functional MRI data [20,21].

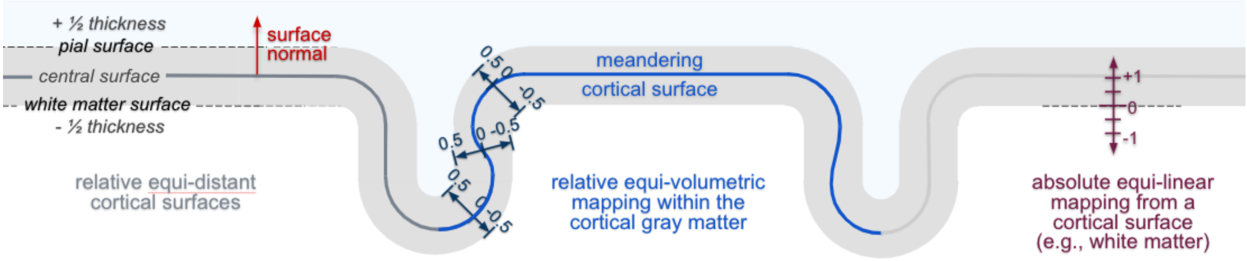

Supplemental Figure 7: Volume mapping. CAT12 offers multiple ways to map voxel values onto the surface. The default mapping extracts voxel values at multiple positions along a surface normal between the white matter surface and the pial surface. The exact location of these positions along the normal is determined by an equi-volumetric model [19], which reflects the shift of cortical layers caused by local folding. However, voxel values can also be extracted at a specific user-defined displacement (in mm) from any given surface location.

Supplemental Note 6. Threshold-free Cluster Enhancement (TFCE)

SPM's standard correction for multiple comparisons is based either on the magnitude of the T or F statistic (correction on voxel-level) or on the extent of clusters in a thresholded statistical map (correction on cluster level). The principle of TFCE – as implemented in CAT12's TFCE toolbox – is to combine both approaches, which has several theoretical and practical advantages, as detailed elsewhere [9]. Briefly, it retains the sensitivity of cluster-based inferences, while avoiding their main downsides, such as arbitrary cluster-forming thresholds or susceptibility to non-stationarity that may compromise the statistical validity [22–24]. As a special feature in CAT, the TFCE toolbox automatically recognizes exchangeability blocks and potential nuisance parameters [25], which would otherwise need to be specified by the user.

Supplemental Note 7. Customized Methods for Clinical Data

Stroke Lesion Correction (SLC)

To mitigate improper deformations during spatial registration in brains with stroke lesions, the CAT12 toolbox offers a Stroke Lesion Correction (SLC) method. This feature suppresses strong (high-frequency) deformations during the Shooting registration step, which can occur due to the presence of lesions. To utilize this method, the lesions must be set to zero. This can be achieved by employing the Manual Image Masking batch, where a lesion mask can be created. Subsequently, the SLC flag should be enabled in the expert mode of CAT12. This ensures that the regions containing lesions are excluded from the spatial registration, preventing large deformations that might otherwise arise when aligning the lesioned brain with a template brain. By implementing this correction, CAT12 facilitates more accurate spatial alignment, particularly for clinical data involving stroke patients. This approach is essential for neuroimaging studies, where a precise alignment of brain structures is crucial for the subsequent analysis.

White Matter Hyperintensity Correction (WMHC)

The accurate detection of white matter hyperintensities (WMHs) is crucial to prevent registration errors, such as the inappropriate mapping of WMHs to typical gray matter locations. Additionally, WMHs in close proximity to the cortex can lead to surface reconstruction errors by being misinterpreted as gray matter.

To address this issue, CAT12 initially employs a low-resolution shooting registration technique [6] on the preliminary SPM segments to align the tissue probability map and the CAT12 atlas with

the individual image space. Subsequently, local tissue and region corrections are conducted using region-growing and bottleneck algorithms [26].
Within the individual segmentation map, isolated GM islands within the WM and voxels adjacent to the lateral ventricles that have high WM probability but GM-like intensity are classified as WMHs. These areas with GM-like intensity but a WMH label are either temporarily aligned with WM or treated as a separate tissue class, depending on the WMH correction (WMHC) processing parameters.

Supplemental Tables

Supplemental Table 1: Voxel-based ROI atlases available in CAT12 (as of October 2023)

Atlas	Reference
Neuromorphometrics [27]	[27]
LPBA40	[28,29]
Cobra	[30–35]<br>(built from 5 atlases provided by the Computational Brain Anatomy Laboratory at the Douglas Institute)
Mori	[36,37]
IBSR [38]	[38]
Hammers	[39,40]
JuBrain Anatomy	[41,42]
Julich-Brain Cytoarchitectonic Atlas	[43,44]
AAL3	[45–47]
Thalamus	[48,49]
Thalamic Nuclei	[50,51]
Melbourne Subcortical Atlas	[52,53]
SUIT Atlas of the human cerebellum	[54,55]

Supplemental Table 2: Surface-based ROI atlases available in CAT (as of October 2023)

Atlas	Reference
DK40 (Desikan-Killiany)	[56,57]
Destrieux	[58,59]
Human Connectome Project (HCP) Multi-Modal Parcellation	[60,61]
Local-Global Intrinsic Functional Connectivity Parcellation	[62,63]

References

[1] SPM12. http://www.fil.ion.ucl.ac.uk/spm/software/spm12. Accessed 1 Jul 2024

[2] FSL. https://www.fmrib.ox.ac.uk/fsl. Accessed 1 Jul 2024

[3] FreeSurfer. https://surfer.nmr.mgh.harvard.edu. Accessed 1 Jul 2024

[4] CIVET. https://mcin.ca/technology/civet. Accessed 1 Jul 2024

[5] Ashburner J, Friston KJ. Unified segmentation. *NeuroImage*, vol. 26, no. 3, pp. 839–51, 2005, doi: 10.1016/j.neuroimage.2005.02.018.

[6] Ashburner J, Friston KJ. Diffeomorphic registration using geodesic shooting and Gauss-Newton optimisation. *NeuroImage*, vol. 55, no. 3, pp. 954–67, Jan. 2011, doi: 10.1016/J.NEUROIMAGE.2010.12.049.

[7] FSL User Guide. https://fsl.fmrib.ox.ac.uk/fsl/fslwiki/FSLVBM/UserGuide. Accessed 1 Jul 2024

[8] CBRAIN. https://portal.cbrain.mcgill.ca. Accessed 1 Jul 2024

[9] Smith SM, Nichols TE. Threshold-free cluster enhancement: Addressing problems of smoothing, threshold dependence and localisation in cluster inference. *NeuroImage*, vol. 44, no. 1, pp. 83–98, 2009.

[10] BrainWeb. https://brainweb.bic.mni.mcgill.ca/brainweb. Accessed 1 Jul 2024

[11] Ashburner J, Ridgway GR. Symmetric diffeomorphic modeling of longitudinal structural MRI. *Front. Neurosci.*, vol. 6, no. 197, Jan. 2012, doi: 10.3389/FNINS.2012.00197.

[12] Reuter M, Rosas HD, Fischl B. Highly accurate inverse consistent registration: a robust approach. *NeuroImage*, vol. 53, no. 4, pp. 1181–96, Jul. 2010, doi: 10.1016/J.NEUROIMAGE.2010.07.020.

[13] Reuter M, Fischl B. Avoiding asymmetry-induced bias in longitudinal image processing. *NeuroImage*, vol. 57, no. 1, pp. 19–21, Jul. 2011, doi: 10.1016/j.neuroimage.2011.02.076.

[14] Broessner G, Ellerbrock I, Menz MM, Frank F, Verius M, Gaser C, May A. Repetitive T1 Imaging Influences Gray Matter Volume Estimations in Structural Brain Imaging. *Front. Neurol.*, vol. 12, p. 755749, Oct. 2021, doi: 10.3389/fneur.2021.755749.

[15] Taubert M, Mehnert J, Pleger B, Villringer A. Rapid and specific gray matter changes in M1 induced by balance training. *NeuroImage*, vol. 133, pp. 399–407, Jun. 2016, doi: 10.1016/j.neuroimage.2016.03.017.

[16] Gilmore AD, Buser NJ, Hanson JL. Variations in structural MRI quality significantly impact commonly used measures of brain anatomy. *Brain Inform.*, vol. 8, no. 7, Apr. 2021, doi: 10.1186/S40708-021-00128-2.

[17] Dahnke R, Gaser C. Surface and Shape Analysis. in *Brain Morphometry*, Springer New York, 2018, pp. 51–73. doi: 10.1007/978-1-4939-7647-8_4.

[18] Tucholka A, Fritsch V, Poline J-B, Thirion B. An empirical comparison of surface-based and volume-based group studies in neuroimaging. *NeuroImage*, vol. 63, no. 3, pp. 1443–1453, Nov. 2012, doi: 10.1016/j.neuroimage.2012.06.019.

[19] Bok ST. Der Einfluss der in den Furchen und Windungen auftretenden Krümmungen der Grosshirnrinde auf die Rindenarchitektur. *Eur. Arch. Psychiatry Clin. Neurosci.*, vol. 121, no. 1, pp. 682–750, Dec. 1929, doi: 10.1007/BF02864437.

[20] Kemper VG, Martino FD, Emmerling TC, Yacoub E, Goebel R. High resolution data analysis strategies for mesoscale human functional MRI at 7 and 9.4T. *NeuroImage*, vol. 164, pp. 48–58, Apr. 2017, doi: 10.1016/J.NEUROIMAGE.2017.03.058.

[21] Waehnert MD, Dinse J, Weiss M, Streicher MN, Waehnert P, Geyer S, Turner R, Bazin P-L. Anatomically motivated modeling of cortical laminae. *NeuroImage*, vol. 93 Pt 2, pp. 210–20, Apr. 2013, doi: 10.1016/J.NEUROIMAGE.2013.03.078.

[22] Eklund A, Nichols TE, Knutsson H. Cluster failure: Why fMRI inferences for spatial extent have inflated false-positive rates. *Proc. Natl. Acad. Sci.*, vol. 113, no. 28, pp. 7900–5, Jun. 2016, doi: 10.1073/PNAS.1602413113.

[23] Hayasaka S, Phan KL, Liberzon I, Worsley KJ, Nichols TE. Nonstationary cluster-size inference with random field and permutation methods. *NeuroImage*, vol. 22, no. 2, pp. 676–87, Jun. 2004, doi: 10.1016/J.NEUROIMAGE.2004.01.041.

[24] Salimi-Khorshidi G, Smith SM, Nichols TE. Adjusting the effect of nonstationarity in cluster-based and TFCE inference. *NeuroImage*, vol. 54, no. 3, pp. 2006–19, Oct. 2010, doi: 10.1016/J.NEUROIMAGE.2010.09.088.

[25] Winkler AM, Ridgway GR, Webster MA, Smith SM, Nichols TE. Permutation inference for the general linear model. *NeuroImage*, vol. 92, pp. 381–97, Feb. 2014, doi: 10.1016/J.NEUROIMAGE.2014.01.060.

[26] Dahnke R, Yotter RA, Gaser C. Cortical thickness and central surface estimation. *NeuroImage*, vol. 65, pp.

336–348, Jan. 2013, doi: 10.1016/j.neuroimage.2012.09.050.

[27] Neuromorphometrics. http://neuromorphometrics.com. Accessed 1 Jul 2024

[28] Shattuck DW, Mirza M, Adisetiyo V, Hojatkashani C, Salamon G, Narr KL, Poldrack RA, Bilder RM, Toga AW. Construction of a 3D probabilistic atlas of human cortical structures. *NeuroImage*, vol. 39, no. 3, pp. 1064–80, Nov. 2007, doi: 10.1016/J.NEUROIMAGE.2007.09.031.

[29] LPBA40 Atlas. http://www.loni.usc.edu/atlases/Atlas_Detail.php?atlas_id=12. Accessed 1 Jul 2024

[30] Cobra. http://cobralab.ca. Accessed 1 Jul 2024

[31] Amaral RSC, Park MTM, Devenyi GA, Lynn V, Pipitone J, Winterburn J, Chavez S, Schira M, Lobaugh NJ, Voineskos AN, Pruessner JC, Chakravarty MM, Initiative ADN. Manual segmentation of the fornix, fimbria, and alveus on high-resolution 3T MRI: Application via fully-automated mapping of the human memory circuit white and grey matter in healthy and pathological aging. *NeuroImage*, vol. 170, pp. 132–150, Oct. 2016, doi: 10.1016/J.NEUROIMAGE.2016.10.027.

[32] Park MTM, Pipitone J, Baer LH, Winterburn JL, Shah Y, Chavez S, Schira MM, Lobaugh NJ, Lerch JP, Voineskos AN, Chakravarty MM. Derivation of high-resolution MRI atlases of the human cerebellum at 3T and segmentation using multiple automatically generated templates. *NeuroImage*, vol. 95, pp. 217–31, Mar. 2014, doi: 10.1016/J.NEUROIMAGE.2014.03.037.

[33] Treadway MT, Waskom ML, Dillon DG, Holmes AJ, Park MTM, Chakravarty MM, Dutra SJ, Polli FE, Iosifescu DV, Fava M, Gabrieli JDE, Pizzagalli DA. Illness progression, recent stress, and morphometry of hippocampal subfields and medial prefrontal cortex in major depression. *Biol. Psychiatry*, vol. 77, no. 3, pp. 285–294, Jul. 2014, doi: 10.1016/J.BIOPSYCH.2014.06.018.

[34] Tullo S, Devenyi GA, Patel R, Park MTM, Collins DL, Chakravarty MM. Warping an atlas derived from serial histology to 5 high-resolution MRIs. *Sci. Data*, vol. 5, no. 180107, Jun. 2018, doi: 10.1038/SDATA.2018.107.

[35] Winterburn JL, Pruessner JC, Chavez S, Schira MM, Lobaugh NJ, Voineskos AN, Chakravarty MM. A novel in vivo atlas of human hippocampal subfields using high-resolution 3 T magnetic resonance imaging. *NeuroImage*, vol. 74, pp. 254–65, Feb. 2013, doi: 10.1016/J.NEUROIMAGE.2013.02.003.

[36] Mori Atlas. http://wiki.slicer.org/slicerWiki/index.php/Slicer3:Mori-Atlas_labels. Accessed 1 Jul 2024

[37] Oishi K, Faria A, Jiang H, Li X, Akhter K, Zhang J, Hsu JT, Miller MI, Van Zijl P, Albert M. Atlas-based whole brain white matter analysis using large deformation diffeomorphic metric mapping: application to normal elderly and Alzheimer’s disease participants. *Neuroimage*, vol. 46, pp. 486–99, 2009, doi: 10.1016/j.neuroimage.2009.01.002.

[38] IBSR. http://www.nitrc.org/projects/ibsr. Accessed 1 Jul 2024

[39] Hammers A, Allom R, Koepp MJ, Free SL, Myers R, Lemieux L, Mitchell TN, Brooks DJ, Duncan JS. Three-dimensional maximum probability atlas of the human brain, with particular reference to the temporal lobe. *Hum. Brain Mapp.*, vol. 19, pp. 224–247, Aug. 2003.

[40] Hammers Atlas. http://brain-development.org/brain-atlases/adult-brain-atlases/individual-adult-brain-atlases-new. Accessed 1 Jul 2024

[41] Eickhoff SB, Stephan KE, Mohlberg H, Grefkes C, Fink GR, Amunts K, Zilles K. A new SPM toolbox for combining probabilistic cytoarchitectonic maps and functional imaging data. *NeuroImage*, vol. 25, no. 4, pp. 1325–35, May 2005, doi: 10.1016/J.NEUROIMAGE.2004.12.034.

[42] JuBrain Atlas. https://github.com/inm7/jubrain-anatomy-toolbox

[43] Amunts K, Mohlberg H, Bludau S, Zilles K. Julich-Brain: A 3D probabilistic atlas of the human brain’s cytoarchitecture. *Science*, vol. 369, no. 6506, pp. 988–992, Jul. 2020, doi: 10.1126/SCIENCE.ABB4588.

[44] Julich Brain Atlas. https://kg.ebrains.eu/search/instances/Dataset/3fde2768-e845-4fc3-a425-61e2c1fb6db7. Accessed 1 Jul 2024

[45] Rolls ET, Huang C-C, Lin C-P, Feng J, Joliot M. Automated anatomical labelling atlas 3. *NeuroImage*, vol. 206, no. 116189, Sep. 2019, doi: 10.1016/J.NEUROIMAGE.2019.116189.

[46] Tzourio-Mazoyer N, Landeau B, Papathanassiou D, Crivello F, Etard O, Delcroix N, Mazoyer B, Joliot M. Automated anatomical labeling of activations in SPM using a macroscopic anatomical parcellation of the MNI MRI single-subject brain. *NeuroImage*, vol. 15, no. 1, pp. 273–89, Jan. 2002, doi: 10.1006/NIMG.2001.0978.

[47] AAL Atlas. http://www.gin.cnrs.fr/en/tools/aal. Accessed 1 Jul 2024

[48] Najdenovska E, Alemán-Gómez Y, Battistella G, Descoteaux M, Hagmann P, Jacquemont S, Maeder P, Thiran

J-P, Fornari E, Cuadra MB. In-vivo probabilistic atlas of human thalamic nuclei based on diffusion- weighted magnetic resonance imaging. *Sci. Data*, vol. 5, no. 180270, Nov. 2018, doi: 10.1038/SDATA.2018.270.

[49] Thalamus Nuclei Atlas. https://wp.unil.ch/mial/probabilistic-atlas-of-thalamic-nuclei

[50] Saranathan M, Iglehart C, Monti M, Tourdias T, Rutt B. In vivo high-resolution structural MRI-based atlas of human thalamic nuclei. *Sci. Data*, vol. 8, no. 275, Oct. 2021, doi: 10.1038/S41597-021-01062-Y.

[51] Thalamic Nuclei Atlas. https://github.com/thalamicseg/thomas_new. Accessed 1 Jul 2024

[52] Tian Y, Margulies DS, Breakspear M, Zalesky A. Topographic organization of the human subcortex unveiled with functional connectivity gradients. *Nat. Neurosci.*, vol. 23, no. 11, pp. 1421–1432, Sep. 2020, doi: 10.1038/S41593-020-00711-6.

[53] Melbourne Subcortical Atlas. https://github.com/yetianmed/subcortex

[54] Diedrichsen J, Balsters J, Flavell J, Cussans E, Ramnani N. A probabilistic MR atlas of the human cerebellum. *NeuroImage*, vol. 47, no. S122, Jul. 2009, doi: 10.1016/S1053-8119(09)71166-8.

[55] SUIT Atlas. https://github.com/DiedrichsenLab/cerebellar_atlases. Accessed 1 Jul 2024

[56] Desikan RS, Ségonne F, Fischl B, Quinn BT, Dickerson BC, Blacker D, Buckner RL, Dale AM, Maguire RP, Hyman BT, Albert MS, Killiany RJ. An automated labeling system for subdividing the human cerebral cortex on MRI scans into gyral based regions of interest. *NeuroImage*, vol. 31, no. 3, pp. 968–80, 2006, doi: DOI: 10.1016/j.neuroimage.2006.01.021.

[57] DK40 Atlas. https://surfer.nmr.mgh.harvard.edu/fswiki/CorticalParcellation. Accessed 1 Jul 2024

[58] Destrieux C, Fischl B, Dale A, Halgren E. A sulcal depth-based anatomical parcellation of the cerebral cortex. *NeuroImage*, vol. 47, Jul. 2009, doi: 10.1016/S1053-8119(09)71561-7.

[59] Destrieux Atlas. https://surfer.nmr.mgh.harvard.edu/fswiki/DestrieuxAtlasChanges. Accessed 1 Jul 2024

[60] Glasser MF, Coalson TS, Robinson EC, Hacker CD, Harwell J, Yacoub E, Ugurbil K, Andersson J, Beckmann CF, Jenkinson M. A multi-modal parcellation of human cerebral cortex. *Nature*, 2016; 536, pp. 171–178

[61] HCP Atlas. https://balsa.wustl.edu/study/RVVG. Accessed 1 Jul 2024

[62] Schaefer A, Kong R, Gordon EM, Laumann TO, Zuo X-N, Holmes AJ, Eickhoff SB, Yeo BTT. Local-Global Parcellation of the Human Cerebral Cortex from Intrinsic Functional Connectivity MRI. *Cereb. Cortex*, vol. 28, no. 9, pp. 3095–3114, Sep. 2018, doi: 10.1093/CERCOR/BHX179.

[63] Local-Global Atlas. https://github.com/ThomasYeoLab/CBIG/tree/master/stable_projects/brain_parcellation/Schaefer2018_LocalGlobal. Accessed 1 Jul 2024
