# Supplementary material for: CAT: a computational anatomy toolbox for the analysis of structural MRI data
Source: Gigascience. 2024 Aug 5;13:giae049. doi: 10.1093/gigascience/giae049 (PMC11299546; doi:10.1093/gigascience/giae049)
Supplement: giae049_Response_to_Reviewer_Comments_Original_Submission [file giae049_response_to_reviewer_comments_original_submission.pdf]

Author's Response To Reviewer Comments

Reviewer reports:
Reviewer #1: This Technical Note describes the Computational Anatomy Toolbox (CAT) software tool, which includes a Graph
MRI data. The CAT software tool is impressive, and enables voxel-based and surface-based morphometric analysis to be acco
and surface mesh generation to be applied to these 3D imaging datasets. The authors helpfully illustrate the utility of the Cor
images from the Alzheimer's Disease Neuroimaging Initiative (ADNI) database.

This is an excellent, freely available tool for the Neuroimaging community and the authors are to be commended for developi

Thank you very much.

Minor comments

I first attempted to launch the CAT software tool on macOS 14.0 (Sonoma) with Apple M1 chip, and on the command line I re
You should move it to the Bin.

I additionally tested the CAT software tool on macOS 12.6 (Monterey) with Intel chip, and I was able to run the CAT software

A: Thank you for testing CAT12. We are happy it ran smoothly on macOS 12.6. With respect to the error on macOS 14, pleas
various issues. So, it could be that you got unlucky with the arm64 version. Apologies, this version is no longer available on t
I have tested on my MacOS 14.4 Mac with M1 and M2 processors (it ran smoothly without any problems). If the problem on y
software on Apple silicon processors, is not yet installed. Since your standalone version ran on another computer with an Inte
security system described here:
https://www.fil.ion.ucl.ac.uk/spm/docs/wikibooks/Installation_on_64bit_Mac_OS_%28Intel%29/#macos-catalina-big-sur-mo

A minor criticism is that the installation instructions in the supporting Readme file for archive [CAT12.9_R2023b_MCR_Mac_a
the SPM (Statistical Parametric Mapping) software tool. The CAT software tool needs to be downloaded separately and then n
instructions are included in the supporting CAT software documentation (https://neuro-jena.github.io/cat12-help/#get_starte

A: The aforementioned CAT12 standalone version already contains everything (SPM12 and CAT12), and there is no need to in
standalone version (which is the default use), SPM12 and CAT12 have to be installed separately, which is also described in th

With the issues I encountered in installation, I invite the authors to list the System Requirements - specifically the Operating
manuscript and also in the supporting CAT software documentation.

A: Currently there are no system requirements and CAT12 runs successfully on a variety of different systems without any pro
potential bugs that may occur for some CAT12 versions and computer systems, which also allows us to fix these bugs in a tin
https://www.neuro.uni-
jena.de/piwik/index.php?module=CoreHome&action=index&idSite=1&period=day&date=today#?idSite=1&period=day&date=

In addition, it would be particularly helpful if the instructions on how to install CAT in the context of SPM were included in the
archives.

A: The non-standalone versions contain such a file (README.md), which describes in detail the necessary steps to install CAT
carefully checked the latest standalone versions (from March 20th) and the non-standalone version (from March 8th), and all
had a temporary version (i.e., CAT12.9_R2023b_MCR_Mac_arm64.zip) that I have already removed from the website.

Reviewer #2: Overall, I think the CAT software provides valuable tools to analyse morphometric differences in the brain and
well. However, I think some clarifications would help the readers understand and evaluate the quality of the methods.

Thank you very much.

Comments:
Figure 2: Looking at the chart, I have a question regarding the pipeline. Is it required to run the whole pipeline using CAT? O
analysis or further?

A: No, it is not always required to run the whole pipeline using CAT. For example, we support the use of other segmentation
provided by the reviewers, the spatial registration steps as implemented in CAT12 cannot be bypassed. However, it is possibl
can save these maps (i.e. gray and white matter segmentation) in the (native) space of the original input image.

Voxel-based Processing: The above question is quite important, seeing that the preprocessing uses rather old registration me
especially with clinical populations.

A: We understand the reviewer's concerns but believe that CAT12 uses up-to-date methods. More specifically, with particular
(Ashburner & Friston, 2011; https://doi.org/10.1016/j.neuroimage.2010.12.049) from the Shooting toolbox of SPM12 which
increasing iterations). Shooting is the successor of the DARTEL registration from SPM12, which already showed quite good ac
https://doi.org/10.1016/j.neuroimage.2008.12.037), but uses smaller deformations to achieve the same or better accuracy c

Spatial Registration and Figure 3: For the registration, how is the registration performing with clinical populations (e.g. stroke
specific disorders.

A: Good point. However, due to space limitations, it was impossible to describe all available features of CAT12. However, for
implemented customized approaches (as described in the manual; https://neuro-jena.github.io/cat12-help/#vox_proc) that w

Stroke Lesion Correction (SLC)
To mitigate improper deformations during spatial registration in brains with stroke lesions, the CAT12 toolbox offers a Stroke
frequency) deformations during the Shooting registration step, which can occur due to the presence of lesions. To utilize this
the Manual Image Masking batch, where a lesion mask can be created. Subsequently, the SLC flag should be enabled in the e
excluded from the spatial registration, preventing large deformations that might otherwise arise when aligning the lesioned b
more accurate spatial alignment, particularly for clinical data involving stroke patients. This approach is essential for neuroim
subsequent analysis.

White Matter Hyperintensity Correction (WMHC)
The accurate detection of white matter hyperintensities (WMHs) is crucial to prevent registration errors, such as the inapprop
in close proximity to the cortex can lead to surface reconstruction issues by being misinterpreted as gray matter (GM). To ad
technique (Ashburner & Friston, 2011) on the preliminary SPM segments to align the tissue probability map and the CAT12 a
corrections are conducted using region-growing and bottleneck algorithms (Dahnke et al., 2013).Within the individual segme
adjacent to the lateral ventricles that have high WM probability but GM-like intensity are classified as WMHs. These areas wit
treated as a separate tissue class, depending on the WMH correction (WMHC) processing parameters.

Surface Registration and Figure 3: What type of noise is used to evaluate the accuracy? This can be important as not every n
depending on the modality.

A: We have used the BrainWeb Phantom (https://brainweb.bic.mni.mcgill.ca/brainweb), which allows the simulation of Gauss
segmentation). However, the implemented spatially adaptive non-local means (SANLM) denoising filter (Manjón et al., 2010)

Maybe having the letters of the figure panels referred to in the text would help the reader.

A: We have now added the letters of the figure panels to the text.

Performance of CAT: Although I see the advantage of using simulated data, I think it would require more explanation. First, w
compare to real data? Second, is it only healthy data? In that case, the accuracy evaluation might not be relevant for the ma

A: For our evaluation, we used the BrainWeb Simulated Brain Database, which provides simulated data for normal brains as w
normal brains because our approach relies only on T1-weighted images, whereas the detection of MS lesions requires additio
simulated data, we have used real clinical data from patients with Alzheimer's disease. This enabled us to evaluate the perfor
common neuroimaging tools.

Longitudinal Processing: Are VBM analyses sensitive enough to capture changes over days? I would be surprised, but I would

from it, I reckon).

A: Yes, definitely. VBM analyses are sensitive enough, and there are a number of studies that detected significant changes in
detecting short-term changes after only a few hours! We have added the references (see below) to these latter studies to the

Taubert et al. 2016: Rapid and specific gray matter changes in M1 induced by balance training
https://doi.org/10.1016/j.neuroimage.2016.03.017

Broessner et al. 2021: Repetitive T1 Imaging Influences Gray Matter Volume Estimations in Structural Brain Imaging
https://doi.org/10.3389/fneur.2021.755749

Mapping onto the Cortical Surface: I am a bit confused about the interest in mapping functional or diffusion parameters to th
would waste a lot of information from these parameters, but I am not familiar with this type of analysis.
"Optionally, CAT also allows mapping of voxel values at multiple positions along the surface normal at each node". I do not u

A: Yes, indeed, there are several papers that revolve around mapping onto the cortical surface (e.g., Brodoehl et al., 2020 ht
modalities onto the surface has several advantages, as described in detail in “Supplemental Note 5. Mapping onto the Cortica
in Supplemental Figure 7). Just to give one example: Mapping onto the surface allows for smoothing on the surface using ge
typical smearing across anatomical boundaries that can occur in 3D (Euclidean) space. This, in turn, improves the ability to s
cortex but farther apart in the unfolded cortex.

Example application:
Is there a way to come back from the surface space to the volume space to compare the results? For example, VBM and SBM
they are not in the same space. Additionally, in the end, the surface representation is just that, a representation; most other
translate the result on the surface back to the volume (if it is not already available).

A: In theory, interpolation may allow for the mapping of surface data back to 3D (volume) space, but this is not done in prac
mapping exists. The issue is further complicated by different measures. For example, vertex-wise cortical thickness is a meas
based gray matter quantifies the local amount of tissue in a given voxel.

Evaluation of CAT12:
I was confused with Supplemental Figure 1 as it is not mentioned in the caption that it is the AD data and not the simulated

A: We have now added this information to the figure caption.

Regarding the reliability of CAT12, it seems to capture more things, but I struggle to see how we can be sure that this is "bet

A: While we cannot fully eliminate the possibility of false positives, we have taken measures to minimize this risk. Specificall
wise error (FWE) with a threshold of $p < 0.001$, which significantly reduces the likelihood of false positives. Furthermore, our
hypotheses are based on well-established anatomical patterns of the disease.
We acknowledge that increased sensitivity can lead to concerns about specificity, but the larger effect sizes observed with C
Alzheimer's disease. Given this alignment with established patterns, we are confident that the larger effect sizes are not sole

"those achieved based on manual tracing and demonstrated that both approaches produced comparable hippocampal volum
could be misleading.

A: We acknowledge that comparable volumes do not always equate to identical accuracy. However, in neuroimaging researc
accuracy of automated segmentation methods. The study by Khlif et al. (2019) found that CAT12's automated segmentation
through manual tracing, thereby providing a meaningful assessment of accuracy.
The primary goal of segmentation methods is to approximate the true anatomical volumes as closely as possible, and compa
high degree of accuracy. Additionally, manual tracing is inherently variable due to human error, while automated methods of
estimates indicate that CAT12 performs well within this established standard of accuracy.

I think the multiple studies show that CAT12 is as valid as any other tool but I am not sure the argument that it is better is a
relevant morphological change is for a given disease.

A: We acknowledge that determining what constitutes a relevant morphological change for a given disease can be challenging
sensitivity and accuracy in both real and simulated data, supporting our arguments.
1. Performance on Real Data: Multiple studies, including our own, have shown that CAT12 identifies consistent effects in clinic
patterns of disease-related changes, indicating that CAT12 is sensitive to relevant morphological changes.
2. Validation with Simulated Data: In addition to real data, CAT12 has been tested on simulated datasets where the ground t
accuracy, showing that CAT12 performs well under controlled conditions and is more robust against noise and intensity non-u
3. Consistency with Disease Patterns: The morphological changes detected by CAT12 are consistent with known disease patte
While no tool is perfect, the evidence suggests that CAT12 offers improved sensitivity and accuracy, making it a robust choice

Methods:
Statistical Analysis: Why is the FWER correction used for the voxel-wise statistics (which perform many comparisons) and FD
would expect the opposite.

A: The choice of multiple comparison correction method depends on the specific analysis and its characteristics.
1. Voxel-wise Statistics:
• Voxel-wise analyses involve a large number of comparisons, making them prone to type I errors.
• The FWER correction (such as FWE) is conservative and controls the probability of making any type I error across all compa
• This approach is suitable for voxel-wise analyses because it minimizes false positives across the many comparisons being m
2. ROI-wise Statistics:
• ROI-based analyses involve fewer comparisons, typically related to specific anatomical regions of interest.
• The FDR correction controls the expected proportion of type I errors among the rejected hypotheses, which can be more ap
• FDR provides a good balance between controlling for false positives and maintaining power, especially when the number of

In short, voxel-wise analyses have a high potential for type I errors due to the large number of comparisons, warranting a m
involve fewer comparisons, making FDR a more suitable choice for balancing control of type I error and statistical power.

"The outcomes of the VBM and voxel-based ROI analyses were overlaid onto orthogonal sections of the mean brain created fr

A: We have extended this sentence to "The outcomes of the VBM and voxel-based ROI analyses were overlaid onto orthogona
T1-weighted images of the study sample (n=50)."

Reviewer #3: CAT has been around for a long time and is a well maintained toolbox - the paper describes all the features and
comments on the pdf (uploaded) which I don't see has mandatory and thus 'accepted' the paper (and leave the authors to de
toolbox.

Dr Cyril Pernet

A: Many thanks for your helpful comments in the PDF, which we have considered in the manuscript.
