# Supplementary material for: CAT: a computational anatomy toolbox for the analysis of structural MRI data
Source: Gigascience. 2024 Aug 5;13:giae049. doi: 10.1093/gigascience/giae049 (PMC11299546; doi:10.1093/gigascience/giae049)
Supplement: giae049_Response_to_Reviewer_Comments_Revision_2 [file giae049_response_to_reviewer_comments_revision_2.pdf]

Author's Response To Reviewer Comments

I hope that we have now everything included from your requests.
