# Supplementary material for: CAT: a computational anatomy toolbox for the analysis of structural MRI data
Source: Gigascience. 2024 Aug 5;13:giae049. doi: 10.1093/gigascience/giae049 (PMC11299546; doi:10.1093/gigascience/giae049)
Supplement: giae049_Reviewer_1_Report_Original_Submission — Chris Armit -- 3/21/2024 Reviewed [file giae049_reviewer_1_report_original_submission.pdf]

Reviewer Report

Title: CAT â€“ A Computational Anatomy Toolbox for the Analysis of Structural MRI Data

Version: Original Submission **Date:** 3/21/2024

Reviewer name: Chris Armit

Reviewer Comments to Author:

This Technical Note describes the Computational Anatomy Toolbox (CAT) software tool, which includes a Graphical User Interface that can be used for morphometric analysis of Structural MRI data. The CAT software tool is impressive, and enables voxel-based and surface-based morphometric analysis to be accomplished on Structural MRI data, and also voxel-based tissue segmentation and surface mesh generation to be applied to these 3D imaging datasets. The authors helpfully illustrate the utility of the Computational Anatomy Toolbox (CAT) using T1-weighted structural brain images from the Alzheimer's Disease Neuroimaging Initiative (ADNI) database.This is an excellent, freely available tool for the Neuroimaging community and the authors are to be commended for developing this impressive software tool.Minor commentsI first attempted to launch the CAT software tool on macOS 14.0 (Sonoma) with Apple M1 chip, and on the command line I received the following message: "spm12" is damaged and can't be opened. You should move it to the Bin.I additionally tested the CAT software tool on macOS 12.6 (Monterey) with Intel chip, and I was able to run the CAT software tool on this platform.A minor criticism is that the installation instructions in the supporting Readme file for archive [CAT12.9_R2023b_MCR_Mac_arm64.zip], which runs on macOS with Intel chip, only details how to install the SPM (Statistical Parametric Mapping) software tool. The CAT software tool needs to be downloaded separately and then moved into the directory of the SPM toolbox, and these installation instructions are included in the supporting CAT software documentation (https://neuro-jena.github.io/cat12-help/#get_started)With the issues I encountered in installation, I invite the authors to list the System Requirements - specifically the Operating Systems that are needed to run the CAT software tool - in the GigaScience manuscript and also in the supporting CAT software documentation.In addition, it would be particularly helpful if the instructions on how to install CAT in the context of SPM were included in the supporting Readme files for the Computational Anatomy Toolbox (CAT) zip archives.

Level of Interest

Please indicate how interesting you found the manuscript: Choose an item.

Quality of Written English

Please indicate the quality of language in the manuscript: Choose an item.

Declaration of Competing Interests

Please complete a declaration of competing interests, considering the following questions:

- Have you in the past five years received reimbursements, fees, funding, or salary from an organisation that may in any way gain or lose financially from the publication of this manuscript, either now or in the future?
- Do you hold any stocks or shares in an organisation that may in any way gain or lose financially from the publication of this manuscript, either now or in the future?
- Do you hold or are you currently applying for any patents relating to the content of the manuscript?
- Have you received reimbursements, fees, funding, or salary from an organization that holds or has applied for patents relating to the content of the manuscript?
- Do you have any other financial competing interests?
- Do you have any non-financial competing interests in relation to this paper?

If you can answer no to all of the above, write 'I declare that I have no competing interests' below. If your reply is yes to any, please give details below.

I work for GigaScience Press where I perform checks on the quality of imaging data. I declare that I have no competing interests, and my funding is not dependent on the outcome of this review.

I agree to the open peer review policy of the journal. I understand that my name will be included on my report to the authors and, if the manuscript is accepted for publication, my named report including any attachments I upload will be posted on the website along with the authors' responses. I agree for my report to be made available under an Open Access Creative Commons CC-BY license (http://creativecommons.org/licenses/by/4.0/). I understand that any comments which I do not wish to be included in my named report can be included as confidential comments to the editors, which will not be published.

Choose an item.

To further support our reviewers, we have joined with Publons, where you can gain additional credit to further highlight your hard work (see: https://publons.com/journal/530/gigascience). On publication of this paper, your review will be automatically added to Publons, you can then choose whether or not to claim your Publons credit. I understand this statement.

Yes Choose an item.
