# Supplementary material for: CAT: a computational anatomy toolbox for the analysis of structural MRI data
Source: Gigascience. 2024 Aug 5;13:giae049. doi: 10.1093/gigascience/giae049 (PMC11299546; doi:10.1093/gigascience/giae049)
Supplement: giae049_Reviewer_2_Report_Original_Submission — Chris Foulon -- 4/10/2024 Reviewed [file giae049_reviewer_2_report_original_submission.pdf]

Reviewer Report

Title: CAT â€“ A Computational Anatomy Toolbox for the Analysis of Structural MRI Data

Version: Original Submission **Date:** 4/10/2024

Reviewer name: Chris Foulon

Reviewer Comments to Author:

Overall, I think the CAT software provides valuable tools to analyse morphometric differences in the brain and promotes open science. The study shows the software's capabilities rather well. However, I think some clarifications would help the readers understand and evaluate the quality of the methods.Comments:Figure 2: Looking at the chart, I have a question regarding the pipeline. Is it required to run the whole pipeline using CAT? Or is it possible to input already registered data to start directly with the VBM analysis or further?Voxel-based Processing: The above question is quite important, seeing that the preprocessing uses rather old registration methods. The users might want to use more recent registration methods, especially with clinical populations.Spatial Registration and Figure 3: For the registration, how is the registration performing with clinical populations (e.g. stroke patients)? It can be significant for the applicability of the methods with specific disorders.Surface Registration and Figure 3: What type of noise is used to evaluate the accuracy? This can be important as not every noise can be modelled easily, and some noises are more or less pronounced depending on the modality.Maybe having the letters of the figure panels referred to in the text would help the reader.Performance of CAT: Although I see the advantage of using simulated data, I think it would require more explanation. First, what tells the reader the quality of this simulated data, and how does it compare to real data? Second, is it only healthy data? In that case, the accuracy evaluation might not be relevant for the majority of the clinical studies using CAT.Longitudinal Processing: Are VBM analyses sensitive enough to capture changes over days? I would be surprised, but I would be interested to see studies doing it (and the readers would also benefit from it, I reckon).Mapping onto the Cortical Surface: I am a bit confused about the interest in mapping functional or diffusion parameters to the surface. Do you have examples of articles doing that? It sounds like it would waste a lot of information from these parameters, but I am not familiar with this type of analysis."Optionally, CAT also allows mapping of voxel values at multiple positions along the surface normal at each node". I do not understand this sentence; I think it should be clarified.Example application:Is there a way to come back from the surface space to the volume space to compare the results? For example, VBM and SBM should provide fairly similar results, but comparing them is difficult when they are not in the same space. Additionally, in the end, the surface representation is just that, a representation; most other analyses are still done on the volume space, so it could be helpful to translate the result on the surface back to the volume (if it is not already available).Evaluation of CAT12:I was confused with Supplemental Figure 1 as it is not mentioned in the caption that it is the AD data and not the simulated one. Maybe it would help the reader to mention it.Regarding the reliability of CAT12, it seems to capture more things, but I struggle to see how we can be sure that this is "better" than other methods; couldn't it be false positives?"those achieved based on manual tracing and demonstrated that both approaches produced comparable hippocampal

volume." comparable volumes do not really mean the same accuracy; this sentence could be misleading.I think the multiple studies show that CAT12 is as valid as any other tool but I am not sure the argument that it is better is as solid. Of course, I understand that there is no ground truth for what a relevant morphological change is for a given disease.Methods:Statistical Analysis: Why is the FWER correction used for the voxel-wise statistics (which perform many comparisons) and FDR used on ROI-wise statistics (which perform much fewer comparisons)? I would expect the opposite."The outcomes of the VBM and voxel-based ROI analyses were overlaid onto orthogonal sections of the mean brain created from the entire study sample (n=50); " I don't understand what this refers to.

Level of Interest

Please indicate how interesting you found the manuscript: Choose an item.

Quality of Written English

Please indicate the quality of language in the manuscript: Choose an item.

Declaration of Competing Interests

Please complete a declaration of competing interests, considering the following questions:

- Have you in the past five years received reimbursements, fees, funding, or salary from an organisation that may in any way gain or lose financially from the publication of this manuscript, either now or in the future?
- Do you hold any stocks or shares in an organisation that may in any way gain or lose financially from the publication of this manuscript, either now or in the future?
- Do you hold or are you currently applying for any patents relating to the content of the manuscript?
- Have you received reimbursements, fees, funding, or salary from an organization that holds or has applied for patents relating to the content of the manuscript?
- Do you have any other financial competing interests?
- Do you have any non-financial competing interests in relation to this paper?

If you can answer no to all of the above, write 'I declare that I have no competing interests' below. If your reply is yes to any, please give details below.

I declare that I have no competing interests

I agree to the open peer review policy of the journal. I understand that my name will be included on my report to the authors and, if the manuscript is accepted for publication, my named report including any attachments I upload will be posted on the website along with the authors' responses. I agree for my report to be made available under an Open Access Creative Commons CC-BY license

(http://creativecommons.org/licenses/by/4.0/). I understand that any comments which I do not wish to be included in my named report can be included as confidential comments to the editors, which will not be published.

Choose an item.

To further support our reviewers, we have joined with Publons, where you can gain additional credit to further highlight your hard work (see: https://publons.com/journal/530/gigascience). On publication of this paper, your review will be automatically added to Publons, you can then choose whether or not to claim your Publons credit. I understand this statement.

Yes Choose an item.
